# Emerging Materials, Wearables, and Diagnostic Advancements in Therapeutic Treatment of Brain Diseases

**DOI:** 10.3390/bios12121176

**Published:** 2022-12-16

**Authors:** Brindha Ramasubramanian, Vundrala Sumedha Reddy, Vijila Chellappan, Seeram Ramakrishna

**Affiliations:** 1 Department of Mechanical Engineering, Center for Nanofibers & Nanotechnology, National University of Singapore, Singapore 117574, Singapore; 2 Institute of Materials Research and Engineering (IMRE), Agency for Science, Technology and Research (A*STAR), #08-03, 2 Fusionopolis Way, Innovis, Singapore 138634, Singapore

**Keywords:** brain tumor, Alzheimer, tomography, nanocarriers, bioprinting, wearables, machine learning

## Abstract

Among the most critical health issues, brain illnesses, such as neurodegenerative conditions and tumors, lower quality of life and have a significant economic impact. Implantable technology and nano-drug carriers have enormous promise for cerebral brain activity sensing and regulated therapeutic application in the treatment and detection of brain illnesses. Flexible materials are chosen for implantable devices because they help reduce biomechanical mismatch between the implanted device and brain tissue. Additionally, implanted biodegradable devices might lessen any autoimmune negative effects. The onerous subsequent operation for removing the implanted device is further lessened with biodegradability. This review expands on current developments in diagnostic technologies such as magnetic resonance imaging, computed tomography, mass spectroscopy, infrared spectroscopy, angiography, and electroencephalogram while providing an overview of prevalent brain diseases. As far as we are aware, there hasn’t been a single review article that addresses all the prevalent brain illnesses. The reviewer also looks into the prospects for the future and offers suggestions for the direction of future developments in the treatment of brain diseases.

## 1. Introduction

Brain traumas, illnesses, tumours, and neurodegenerative problems are just a few of the several health issues that can impact the brain under the general term “brain illnesses and disorders” [1,2,3,4,5]. The term “brain diseases” is a category of health conditions that are typically transmissible and frequently brought on by outside factors, such as pathogens, microbes, and injuries as opposed to “brain disorders,” which refers to health conditions that are non-transmittable but frequently have genetically heritable traits and are brought on by the disturbance of the healthy structure and the body’s functions because of birth defects or hereditary malfunctions [6,7,8]. The worsening of behavioural, cognitive, and neurological abilities as a result of cerebral activity impairment is one of the most noteworthy characteristics of brain illnesses and disorders [9,10,11,12]. The intricacy and sensitivity of the organ have made it harder to treat these disorders [5,6,12]. Vaccinations can be used to prevent the emergence of some illnesses, such as bacterial and fungal BI, while particular drugs can treat others like neurological disorders if they are caught in their early stages. Many substances, including most medicines, are prevented from entering the brain by the physical, chemical, and electromagnetic barriers [1,6,8,13,14].

The two biggest obstacles to the treatment of brain illnesses and disorders have already been crossing the blood–brain barrier (BBB) and blood–cerebrospinal fluid (CSF) barrier [15,16]. P-Glycoprotein carefully regulates the efflux of materials through the blood–brain barrier (BBB), hence its dysregulation is linked to the development of neurological disorders and tumours. Nanoparticles (NPs) typically range in size from 1 to 100 nm [17,18,19]. Different types of NPs with diverse physicochemical characteristics have so far been created, including metal oxides, polymeric, nano emulsions, solid-lipid NPs, and dendrites and are used for various applications, including textile, energy, and medicine [20,21,22,23,24,25,26,27]. According to reports, rats’ Glycoprotein-mediated phenytoin resistance is suppressed by nanoparticles [3,17,18,28]. Additionally, a recent study demonstrates that drugs are more permeable to the BBB when it is enclosed in solid lipid NPs. Due to their simple transportability over the BBB and special properties including small size, selectivity, low toxicity, biodegradability, and solubility, NPs based therapies have recently become a promising therapeutic for brain illnesses and disorders [17,29,30]. The majority of medications’ cytotoxicity has an impact on how well they work therapeutically. When compared with traditional treatment, NPs cause substantially less brain toxicity. The drug’s bioactivity and selectivity with NPs guarantee efficient delivery to the intended spot. Drugs are included into NPs to significantly improve these properties [3,17,18].

**Figure 1:**
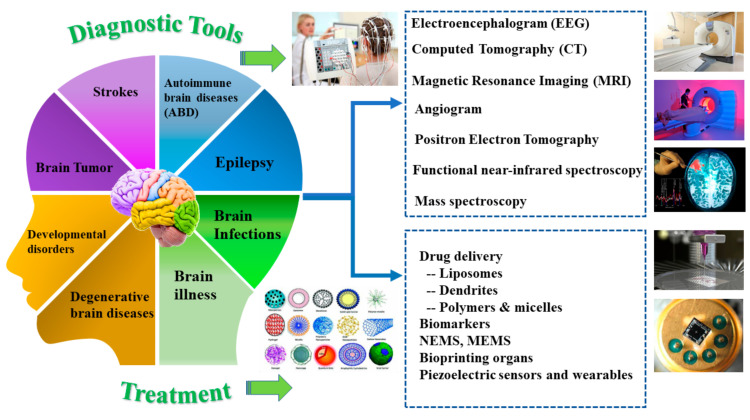
Schematic overview of the review, where the common brain diseases namely, Autoimmune brain diseases, Epilepsy, Brain infections, Brain illness, Neurodegenerative, Neurodevelopmental disorders, brain tumour and strokes, followed by the advancements in the diagnostic tools and treatment approaches highlighting the use of 5G technology, Artificial Intelligence, nanotechnology, self-powered wearables, and microelectromechanical systems.

The background and specifics of prevalent brain disorders are covered In this review, which is followed by developments in therapeutic diagnoses and treatments. To the best of our knowledge, a limited number of papers discuss the combined brain disorders and therapies in straightforward language that can be understood by professionals from different fields all in one place [31,32,33]. The article provides information on how machine learning (ML) and artificial intelligence (AI) are used to enhance diagnostic equipment and medical care [18,32,34,35]. Future possibilities and research directions have been considered in relation to the application of nanotechnology, bioprinting, man-machine interface, self-powering devices, and Micro electrical mechanical systems (MEMS) in the treatment of brain diseases. Figure 1 shows the overview of this article, emphasising the common brain diseases, their impacts, and advancements in diagnosis and treatment. Thus, our article focuses on making the disease and its impacts clear before diving into the diagnosis and treatment. The existing “teach back” method, in which individuals are requested to regurgitate instructions to a health care professional, may not be sufficient to minimize individual errors due to the gap between learning and truly understanding the treatment options without knowing the causes, impacts, and details about the diseases.

## 2. Common Brain Diseases

**Figure 2:**
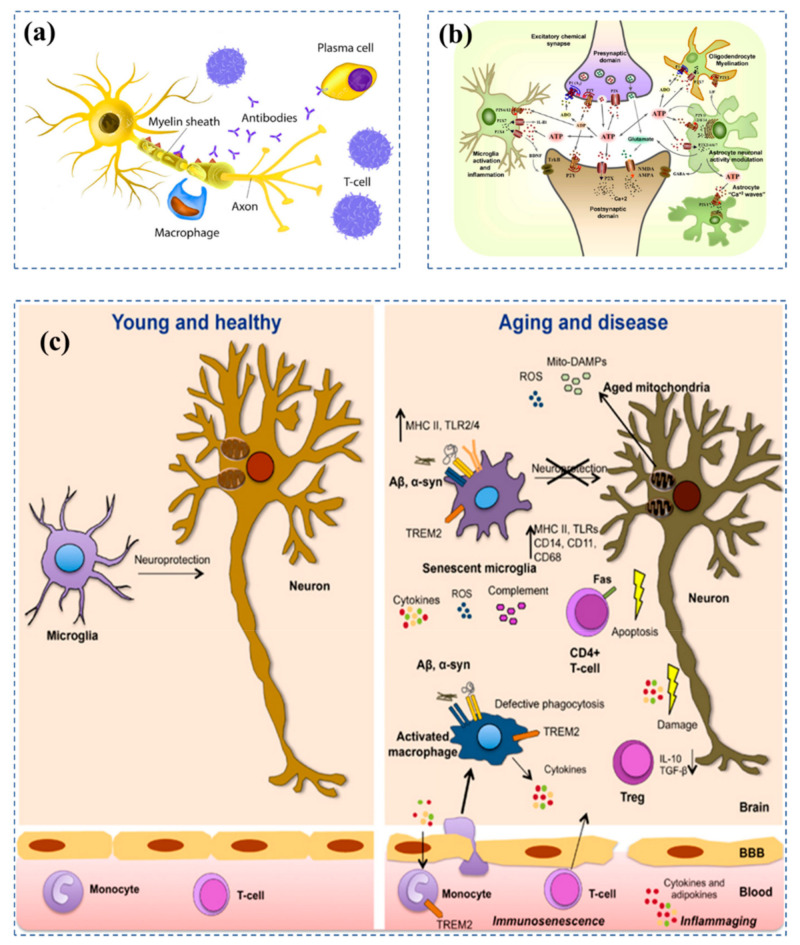
(**a**) Schematic of healthy neuron and brain immune cells (**b**) Microglial AND neuronal receptors in CNS (adapted with permission from [36] Springer Nature, 2008) (**c**) Schematic comparison between healthy neural cells and diseased cells with inflammation (adapted with permission from [37] Frontiers, 2015).

Physical and mental well-being may quickly alter due to autoimmune brain conditions such as autoimmune meningitis and central nervous system (CNS) vasculitis. The brain consists of frontal, parietal, occipital, and temporal lobes in the cerebrum, cerebellum, and brain stem with millions of neurons (the brain’s messenger) and blood vessels as shown in Figure 2. 

There are three primary kinds of glial cells in the brain that support the activity of neurons and maintain brain health; (i) astrocytes facilitate neuronal connectivity, (ii) oligodendrocytes foster quicker information transmission, and (iii) microglia serve as the brain’s immune system [38,39,40]. A vital component of the immune system is the T cell, often known as a T lymphocyte or leukocyte. The schematic of a healthy neuron is shown in Figure 2a. Nerve impulses are created by neurons to communicate with each other. An action potential is a change in the potential electric energy of a neuron brought on by the movement of charged particles into and out of the neuron’s membrane [38,41,42,43,44,45]. A neuron’s terminal has a tiny opening called a synapse, which enables signals to go from one neuron to the next. Axons (Figure 2a) carry action potentials to presynaptic endings once they are formed. These electrical and chemical signals can be sent between neurons at synapses. Presynaptic endings, synaptic clefts, and postsynaptic endings are the components of synapses. Glutamate and ATP are released exocytotically by pre-synaptic terminals as co-transmitters (Figure 2b) [36]. ADP work pre-synaptically to control neurotransmitter release. The P2X4 and P2X7 receptors, which are important in neuropathic pain, are expressed by resting microglia [41,42,46,47,48].

The gradual aging of immune cells occurs in association with the aging of the immune system, as does a protracted inflamed milieu marked by elevated cytokines levels and adipokines (Figure 2c) [37]. These inflammatory chemicals influence neural impulses, stimulate microglia, and susceptible neuronal populations, and they regulate cognitive performance. Upon aging, microglial cells lead to loss of neuroprotective activity and increased susceptibility to atypical inflammatory activation [46,49,50]. The microglia are activated by amyloid build-up and pathogenetic variants of -synuclein, which triggers the production of proinflammatory mediators [51,52,53]. By producing reactive oxygen species (ROS) and damage associated molecular patterns (DAMPs), which activate the NLRP3 receptor inflammasome and immune cells, aged defective mitochondria play a critical role in host defence and age-related inflammation. In turn, dysfunctional mitochondria are exacerbated by inflammatory mediators, and infections encourage a high immunological risk profile (right side of Figure 2c) and the metabolic syndrome [49,50,51,52,53].

### 2.1. Autoimmune Brain Diseases (ABD)

Given that many of the symptoms may be treatable, early identification and treatment are essential for reducing the impact of these conditions. Hereditary factors are one of the primary causes of ABD. Each autoimmune illness is caused by a unique set of genes that have not yet been identified, but several experimental techniques, including genome-wide correlation scans, have been employed to pinpoint specific genetic risk variations that might or may not be at fault [54,55,56]. Researchers are now increasingly understanding the genesis of autoimmune disorders including Type 1 diabetes and rheumatoid arthritis thanks to work on genomic sequencing and hereditary trait transmission studies [49,56,57,58]. As the brain is a complicated organ, studying the impact of genetic changes on ABD poses a number of challenges, not the least of which is the fact that the exact cause of ABD is mostly unclear. The two common ABD are (i) encephalitis and (ii) multiple sclerosis [54,59]. Several brain infections are mis-assumed as ABD. For instance, meningitis is an acute infection of the meninges, the outer membrane surrounding the spinal cord, and brain [60,61,62]. Vasculitis is an inflammation of the walls of blood vessels in the brain and spine [60]. The inflammation arises from the body’s defence system attacking normal tissue and cells in the central nervous system, which causes autoimmune brain disorders. The decreased functionality of the brain brought on by this inflammation may result in psychological problems [63,64]. The wide range of ailments makes diagnosis difficult and frequently delayed for the brain related diseases [54,57,59,65].

Due to the resemblance in the diagnostic, neuroimaging, and laboratory findings of many kinds of autoimmune and infected encephalitis, autoimmune encephalitis is challenging to diagnose clinically [7,66,67]. Patients often experience memory loss and cognitive decline over a few weeks or months. The examination and history may reveal hints about reasons, although frequently such indicators are not present [59]. The right diagnosis can be made using a thorough testing strategy for viral illnesses and different neuronal auto antibodies [68]. When an obvious autoimmune origin of the symptoms is found, the immunological treatments are often intensified [69,70]. To establish the appropriate level of immune treatment required at any given point during the process of care for these individuals, patience and frequent examinations are required [71,72]. Numerous disease forms with various pathophysiologies are included in autoimmune encephalitis [54,59,73,74]. Utilizing medical testing and selecting the best treatments benefit from understanding the pathophysiology of these disorders. The first category consists of the traditional rapidly progressive diseases linked to antibodies, including anti-Hu [75]. These conditions entail T-cell actions that target neurons and have a high cancer association. The severity of the accompanying tumours, the inability to manage certain types of immune responses, and the permanent neuronal death caused by these processes all contribute to the dismal prognosis [54,75,76]. These illnesses’ antibodies serve as helpful diagnostic markers and, depending on the situation and intensity, are also beneficial indicators of paraneoplastic neurological problems. The antibodies do not directly cause disease. Autoimmune reactions to external epitopes of signalling pathways, receptors, and other related proteins, such as the NMDA receptor, make up the second category. The survival is often significantly better, and the cancer correlations can vary. It is believed that the autoantibodies that cause these illnesses are primarily pathogenic and have temporary effects on synaptic function in nerves with minimal loss of dopaminergic neurons [54,57,77,78].

Early in the progression of autoimmune encephalitis, psychiatric symptoms are frequent [76,79]. These include exhilaration, fear, violence, increased anxiety, obsessive behaviours, delusion, and abnormal sexual conduct [49,75,80]. Anti–α-amino-3-hydroxy-5-methyl-4-isoxazolepropionic acid (Anti-AMPAR) receptor and anti-gamma aminobutyric acid-B (Anti-GABA) receptor both have the potential to produce strong early mental signs, although Anti-N-methyl D-aspartate (Anti-NMDAR) receptor encephalitis is widely known for having higher symptoms [57,81,82]. Overall, anti-NMDAR encephalitis is more frequent and should be suspected initially; this is true for young adults and children, though they can both induce similar appearances in people of all ages. Wriggling motions of the face and limbs may be particularly pronounced in the early stages of anti-NMDAR encephalitis in adults. Stiff person syndrome (SPS) or chronic encephalomyelitis with rigid and myoclonic seizures are two possible symptoms of Glutamic acid decarboxylase (GAD65) and Glycine receptor (GlyR) [79,82,83,84]. The majority of autoimmune causes of encephalitis are paraneoplastic, and each has a risk profile for different malignancies [85,86]. Therefore, finding certain tumours may also point to specific immunological mechanisms. For instance, ovarian teratoma is more likely to develop anti-NMDAR encephalitis, while individuals with cognitive degeneration and Hodgkin’s disease are more likely to develop anti-Delta/Notch Like EGF repeat containing protein (anti-DNER) [80,83,84].

The most prevalent neurogenic illness, multiple sclerosis (MS), also known as encephalomyelitis disseminata, causes disruption to the protective coverings of the brain’s nerve cells and spinal cord [87]. This harm interferes with the nervous system’s capacity to transfer messages, leading to various physiological, cognitive, and occasionally behavioural issues as signs and symptoms [59,88,89,90]. While the precise reason is uncertain, the primary possibilities are immune system attack or dysfunction of the myelin-producing cells. ABD may be understood clearly and treated well by excluding other medical causes, including other organ autoimmune illnesses and viral infections [59,89,91].

### 2.2. Epilepsy

Epilepsy is a recurrent, noncommunicable brain disorder that affects over 40 million people worldwide [92,93,94,95]. Its distinctive characteristic is recurrent seizures [79]. Short bursts of impulsive activity known as seizures can sometimes be followed by memory loss and loss of control over one’s body [96,97]. They can affect any part of the body [93,98,99]. Seizures happen as a result of abnormal waves in a group of brain cells [95,100]. Many cerebral regions of the brain are capable of experiencing such discharges [79,96]. Seizures can be caused by even the tiniest muscle twitches or cognitive impairments, as well as by powerful spasms that linger for a long period [79,92]. Seizures might happen less frequently fewer than one per year or more frequently more than one per day [96,99,101,102,103].

The idea that distinct parts of the brain govern various processes is another crucial one for understanding epilepsy [93,94,97]. The earliest symptoms of a seizure that originate from a particular part of the brain frequently correspond to that part’s functions. The left side of the body is managed by the right side of the brain and vice versa. The left thumb or finger may tremble at the commencement of a seizure if it originates on the right side of the brain in the region that regulates thumb motion. The frontal hemispheres, often known as the left and right halves of the cerebrum, are situated in the upper brain. These are connected via the corpus callosum, a group of nerve fibers. Each cerebral hemisphere contains the frontal, parietal, occipital, and temporal lobes [96,102,103,104]. Each lobe has several unique regions that control various capabilities. The brainstem, which is connected to the spinal cord and is situated in the bottom section of the brain, controls breathing, heart rate, and sleep–wake cycles. In the upper part of the brainstem are the hippocampus and hypothalamus. The lower part of the brainstem is carried on by the spinal cord, which relays messages from the brain to the rest of the body. Nerve cells, often known as neurons, are the basic building blocks of the brain [93,94,97,98,104].

In certain situations, the loss of nerve cells might lead to the onset of epilepsy. In the hippocampus, for instance, a sustained shortage of oxygen may result in a progressive destruction of cells, which may induce epilepsy [96]. Some of the brain’s most important neurotransmitters reduce or stop the electrical signalling, which can be measured using electroencephalography (EEG). The reduced signalling stops the activation of nerve cells. These neurotransmitters are referred to as “suppressive” as they prevent cell activity. The most common example of this class is a neurotransmitter known as GABA. In other words, neurotransmitters trigger nerve cell activation [105]. Glutamate is an example of this “excitatory” type. One theory holds that an imbalance between suppressive and excitatory neurotransmitters is the root cause of epilepsy. Several of the novel epilepsy medications in development aim to regulate these neurotransmitters performance. Researchers try to make the excitatory ones, which turn cells on, less active or make the suppressive ones, which turn cells off, active. In either case, the goal is to have less spasms and seizures because of less spontaneous cerebral electrical activity [106,107,108].

**Figure 3:**
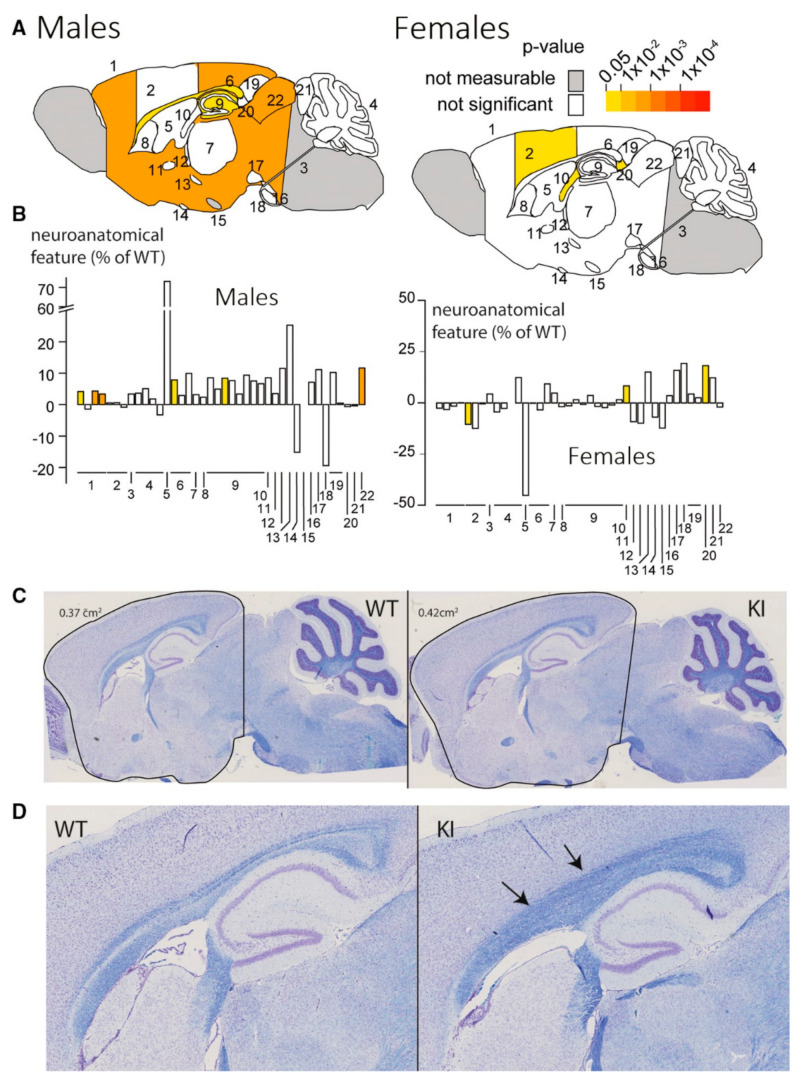
(**A**–**D**) Schematic illustration of brain morphology of male and female mice tested for Kcnq2^T274M/+^ genotype (adapted with permission from [92], Wiley, 2022).

For instance, Mathieu et al. studied the neuro-morphological characteristics of Kcnq2^Thr274Met/+^ mice at 15 weeks of age. The phenotypic knock-in mice were shown to have mild macrocephaly in males with a general trend for regions to be greater in size as compared to wild type mice (Figure 3A,B) [92]. As a result, male mice had a superior colliculus that was 12% bigger and the corpus callosum area that was 8% larger (Figure 3C,D). Several measurements inside the hippocampus also revealed a propensity and even a substantial lengthening in some regions. Little difference between female brains and those of wild animals was seen. These findings suggest that although there are minor gender-specific changes, there are no significant brain morphological impairments in the Kcnq2^Thr274Met/+^ mice [92].

### 2.3. Brain Infections

**Figure 4:**
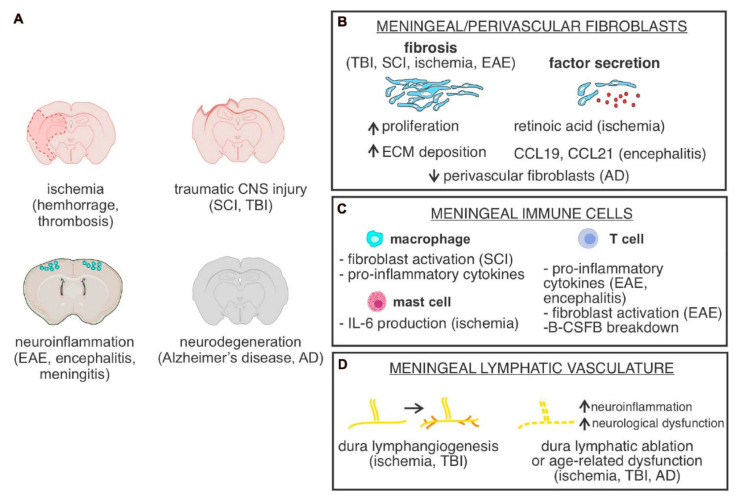
Meningeal tissues or cells in CNS illness or damage (adapted with permission from [108] Frontiers, 2021). (**A**) a CNS injury or illness. (**B**) fibrosis, which is brought on by enhanced perivascular cell growth and extracellular (ECM) accumulation. (**C**) the summary of the functional contributions made by immune cells situated in the meninges in various CNS pathological conditions. (**D**) the summary of the cellular alterations that have occurred in the meninges’ lymphoid system in response to various CNS diseases and traumas.

Any illness that damages the brain or spinal cord through microorganisms like viruses, bacteria, fungi, or parasites is called a brain infection. Brain infections are dangerous and may be fatal. Swelling can result from the immunological activation brought on by illnesses and infections that affect the brain and spinal cord [62,109,110]. Fever, headache, seizures, behavioural abnormalities, and dementia are just a few of the symptoms that these illnesses and the ensuing infection can cause. In severe circumstances, they may cause mortality, head trauma, or a haemorrhage [10,111,112]. There are several kinds of brain infections, and each kind has a particular cause and course of action [2,12,39]. Meningitis caused by bacteria is a dangerous infection that must be treated right away [111]. Occasionally, a virus or fungus can also cause meningitis. Numerous bacteria have the ability to initially produce a respiratory tract infection before moving on to the brain via the blood circulation [109,111]. Additionally, some bacteria can directly enter the meninges and cause bacterial meningitis [111]. Meningitis is often characterized by the abrupt onset of fever, dizziness, neck stiffness, pupil constriction, vomiting, and diarrhoea. Meningitis can be detected if you are not able to lower your head to your chest [10,105,111,113]. It’s possible that the signs initially resemble those associated with a cold or upper respiratory illness, but they can swiftly worsen. For instance, Figure 4A shows the Illustration of a CNS injury or illness in which the illness includes meningeal cells, cell types situated in the meninges, or microvascular fibroblast located in the parenchyma [108]. Here, EAE stands for experimental autoimmune encephalomyelitis. SCI stands for spinal cord damage. Figure 4B shows the graphic representation of fibrosis, which is brought on by enhanced perivascular cell growth and extracellular (ECM) accumulation. Figure 4C shows the summary of the functional contributions made by immune cells situated in the meninges in various CNS pathological conditions. Figure 4D shows the summary of the cellular alterations that have occurred in the meninges’ lymphoid system in response to various CNS diseases and traumas [108].

Several viruses have the potential to attack the central nervous system and adjacent tissues, even if this is somewhat unusual [114,115,116,117]. Herpes simplex, Epstein-Barr, varicella zoster, cytomegalovirus, and influenza viruses are a few examples of potential sources. Zika and other mosquito-borne diseases can cause brain infections as well. Group B Streptococcus, Streptococcus pneumoniae, Haemophilus influenzae, Neisseria meningitidis, and Listeria monocytogenes are most likely to cause a bacterial brain infection [62,110,118]. The Blastomyces, fungi Aspergillus, Coccidioides, Cryptococcus, or Coccidioides can all produce a fungal infection that spreads to the brain. Strongyloides, cysticercosis, toxoplasmosis, and schistosomiasis can all result in a parasite infection of the brain [119,120,121].

One of the most serious conditions is a brain abscess. An abscess may develop when germs from an infection elsewhere in the head, blood circulation, or wound reach the brain. A brain abscess occurs when the surrounding tissue becomes flooded with fluid [119,122,123,124]. Consequently, the tension inside the skull rises, and the muscle around the brain expands [5,8]. The inflammation and pressure increase in proportion to the size of the abscess [125]. Acute meningitis develops if the pus ruptures or bursts, allowing the pus to reach the spinal fluid, which circulates through the tissues covering the spinal cord and central nervous system [80,125,126]. The medical term for an inflammation or infection of the middle ear is otitis media. Ear infections are frequently seen in toddlers, and they can be brought on by both bacteria and viruses [127,128]. This can spread to the spinal fluids and brain [120,124,129,130].

### 2.4. Brain Illness

Mental diseases are ailments that affect emotion, thought, and behavior. Anxiety and/or difficulty coping with daily tasks at work, in the community, or in social situations are symptoms of mental diseases. Some of the common mental illnesses are bipolar disorder, anxiety, depression, schizophrenia, and post-traumatic stress disorder (PTSD) [131,132]. Bipolar illness is assumed to be linked to anomalies in the function and structure of specific brain regions in control of thinking and memory process [133]. According to a neurobiological concept of bipolar illness, the brain’s affective pathway may be separated into two primary groups, Ventral and dorsal system [131,134,135]. The amygdala, insula, ventral striatum, ventral anterior cingulate cortex, and prefrontal cortex are among the brain regions that make up the ventral system, which mediates emotional perception. The hippocampus, dorsal anterior cingulate cortex, and other regions of the prefrontal cortex are all included in the dorsal system, which is in charge of controlling emotions. The ventral system is overactive, and the dorsal system is underactive, according to the model, which suggests that bipolar illness might result from these conditions. According to some models, people’s capacity to control their emotions is hampered [133].

A rise in dopamine causes the homeostatic secondary downregulation of critical system components and receptors, such as the decreased responsiveness of dopamine receptors. Dopamine transport is reduced, which is typical of the depressed phase [60,136,137]. Maintaining homeostasis upregulation might potentially start the cycle afresh after the depressed period. During the bipolar disorder, glutamate is markedly elevated in the left dorso-anterior prefrontal cortex, although levels soon revert to threshold [136,138,139]. Schizophrenia may include hallucinations, illusions, and excessively irrational thought patterns, which can make it difficult to go about daily activities and be incapacitating. Schizophrenia patients require ongoing care [133,140]. Early intervention may help keep the problems under control before major issues arise and it may enhance the prognosis in the long run. The cognitive cycles that impact the links between the thalamus and the cortex are affected by changes in glutamatergic neuronal function, according to the glutamate theory of schizophrenia. Researchers have found that a diminished activation of the NMDA glutamate receptor and drugs like ketamine and phencyclidine can mimic schizophrenia’s characteristics and cognitive issues [139,140]. In addition to anomalies in brain morphometric parameters, post-mortem investigations consistently demonstrate that a portion of these neurons do not express Glutamic acid decarboxylase 67 (GAD67). The aberrant neurons are connected subgroups in schizophrenia, that oversee synchronizing the neuronal ensembles required for tasks involving active memory [132,140,141,142]. These result in gamma impulses, which have a frequency anywhere between 20 and 80 hertz, which are the developed brain cycles. The majority of people who have schizophrenia are not violent and are more inclined to be victims of violence than to commit acts of violence [136]. As part of a larger trend of social isolation, people with schizophrenia are frequently taken advantage of and victims of violent crime. Painful medication treatments, confinement, and constraint are frequently used on people with schizophrenia [141,142,143]. PTSD is a stress condition that can arise after being exposed to very traumatic, frightful, or unpleasant situations. Stress hormone levels in PTSD sufferers are abnormal, according to studies. The body typically creates stress hormones like adrenaline to start a response while in peril [144,145,146]. This response, sometimes referred to as the “flight” response, aids in dulling discomfort and numbing the senses. It has been shown that people with PTSD persist to release large levels of the hormones associated with the fight or flight response even in the absence of a threat. This is assumed to be the cause of the dulled feelings and emotional dysregulation that some PTSD patients encounter [134,147,148,149].

### 2.5. Neurodegenerative Brain Diseases

**Figure 5:**
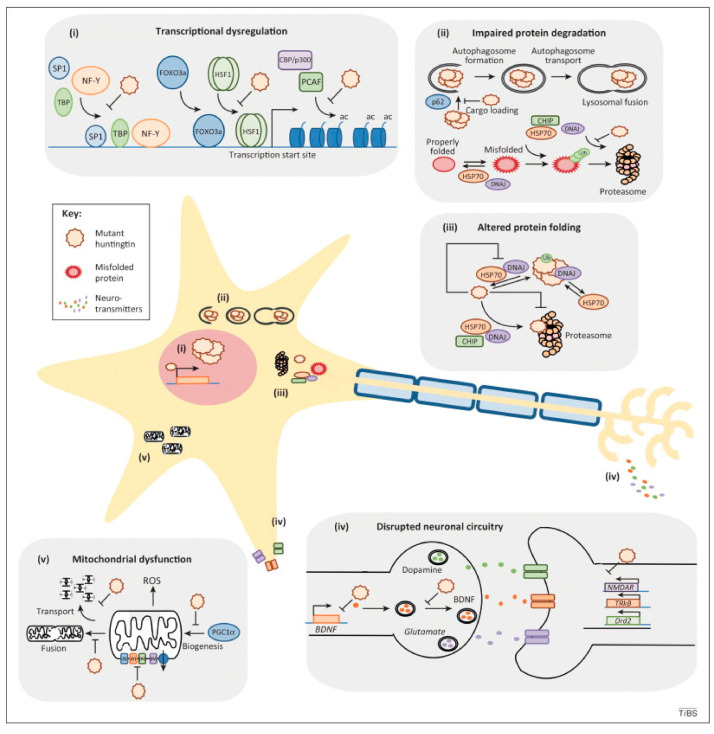
Schematic of significant cellular pathway disruptions causing Huntington’s illness (adapted with permission from [150] Elsevier, 2013).

Whenever nerve cells in the brain begin to lose their functionality over time and eventually fail, this is known as a neurodegenerative illness [150,151,152]. Although certain neurodegenerative disease symptoms can be alleviated with medications, there is presently no curative treatment and just a delay of the illness’s course is possible. Age significantly increases the risk of acquiring a degenerative illness [152,153,154]. Amyotrophic lateral disease, Parkinson’s disease, Alzheimer’s disease, Huntington’s disease, and prion disorders are a few examples of neurodegenerative disorders [154]. Figure 5 shows the (i) transcriptional cognitive deficits of gene expression, (ii) inhibited protein deterioration, (iii) modified protein deformation, (iv) disturbed synaptic signal transduction, and (v) perturbed metabolic activity through modified mitochondrial translation [150]. The researchers depict each route as an independent process; it is likely that disturbances in one pathway affect HD etiology (at least in part) by worsening the dysfunction of other important processes. According to studies, oxidative damage and inflammation are the main causes of neurodegeneration. Alzheimer’s disease (AD) is a long-term neurodegenerative condition that destroys cells in the cerebral cortex. As a result, the occipital cortex, temporal lobes, cerebral lobes, and anterior gyrus all exhibit extensive shrinkage [155]. Amyloid lesions and fibrillary clumps can be observed in the patients with AD. The tiny peptides that make up lesions are known as amyloid beta (Amy-β), and they generally contain 40 to 45 amino acids [156,157]. The loss of dopamine transporters in the midbrain area is the primary feature of Parkinson’s disease (PD). It is unknown what causes this cell death. It is believed that deficiencies in the control of protein transport, including RAB1, may contribute to the pathogenesis of this illness. Studies have shown that alpha-synuclein may be responsible for transmembrane damage [143,156].

Huntington’s disease is a hereditary condition that may be transferred from parents to offspring, in contrast to Parkinson’s disease. This implies that the children are extremely likely to get the sickness if either parent has it [158]. The behavioural condition associated with Huntington’s Disease is brought on by aberrant protein accumulation in the brain, which causes neuronal death [159]. The main symptom of this condition is excessive movement, usually unwelcome and unneeded movement [160]. For instance, people frequently experience uncontrollable limb vibrations that are persistent. People with Huntington’s disease will find it increasingly difficult to move the way they wish to move as the condition worsens. If neurons are dying in any cognitive illness, the brain shrinks, which is a severe situation. An uncommon and deadly recessive neurological condition that first emerges at a young age is batten disease. There are thirteen different lysosomal accumulation diseases together referred to as neuronal ceroid lipofuscinoses (NCLs), among which Batten disease is the popular term. Motor dysfunction, seizures, dementia, eyesight loss, and a shorter life span are the hallmarks of Batten disease [158,159,160].

### 2.6. Neurodevelopmental Disorders

The term “neurodevelopmental disorders” (NDD) refers to a wide range of disorders that affect the development of cognitive or motor skills [161]. Children with NDDs have difficulties with cognitive development, academic success, behavioural, social interactions, and basic traits of daily life [162,163]. In contrast to the remission and relapse cycles seen in different mental diseases, these disorders often manifest with an early onset during childhood, entailing the diminishing or delaying of central nervous system maturation [162]. The discrepancy that exists at various levels, including in the showcase of the condition, the severity of symptoms, the presence of comorbidities, and the appearance of symptoms through time, is the fundamental issue in both diagnosing and treating these disorders [163,164,165,166]. Autism is a paradigmatic NDD since it manifests in infancy, and etiologic research links it to early neurodevelopmental processes that are disrupted and have long-term effects on an individual [161,167]. Developmental disorders are influenced by a variety of genetic, pathophysiological, and environmental factors. Communication and social interaction challenges, repetitive habits, and narrow interests are all features of autism [167]. One of the earliest recognized brain abnormalities in autism was hypoplasia of the posterior vermis, and a key criterion for classifying is decreased cerebellar cortical volume. Severe immune system abnormalities have been observed in autistic people [162,163,168]. It’s possible that autoimmune conditions and anomalies in cytokine production in the body and the brain have an impact on autism [169,170]. Finding more consistent immunological profiles that are only found in this condition could help us better understand its genesis and will be the challenge for the future. In this context, individual variations in autistic traits, including immunological changes, may act as biomarkers for a customized therapeutic strategy [168].

**Figure 6:**
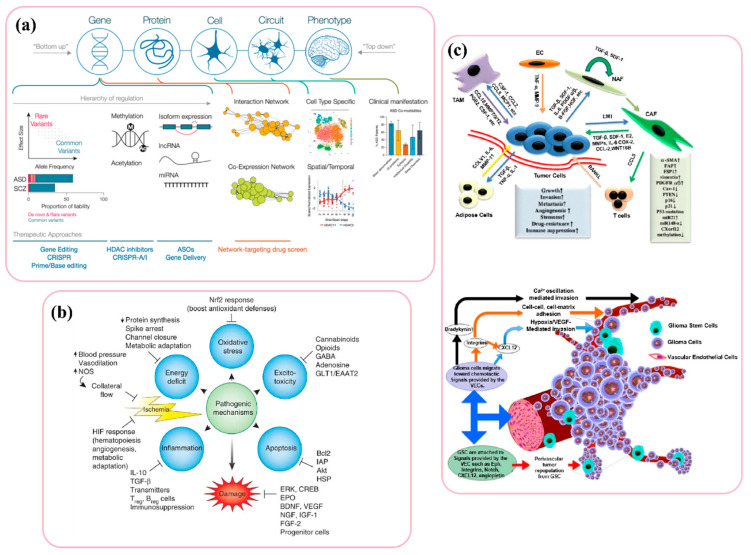
(**a**) Organizational framework for NDD pathogenesis and levels of analysis (adapted with permission from [172] Elsevier, 2022) (**b**) Protective pathways activated by cerebral ischemia, brain stroke (adapted with permission from [173] Springer Nature, 2011) (**c**) Mechanism of interaction of tumor cells with stromal cells in tumor microenvironment promoting tumor growth, invasion, and metastasis (adapted with permission from [174] Springer, 2020).

Despite having characteristics that could place it under the category of disruptive behavioural disorders, schizophrenia is also categorized as an NDD because its causes are increasingly understood to be neurodevelopmental, which is further justified by the large genetic overlap with autism [162,169,170]. Attention-Deficit/Hyperactivity Disorder (ADHD) is characterized by a lack of attention, hyperactivity, and impulsive reaction. The degree of loss in the posterior vermis and overall cerebellar volume has been demonstrated to correspond with the severity of ADHD symptoms [163]. A recent meta-analysis discovered consistent bilateral grey matter reductions in lobule IX corresponding to ADHD [162]. Additionally, it has been noted that there are abnormalities in both anatomical such as middle cerebellar peduncles and functional cerebellar connectivity [168]. Presentation that is predominantly inattentive and presentation that is predominantly hyperactive–impulsive are major categories of ADHD [163,168]. Developmental dyslexia is described as a specialized learning–reading condition that cannot be classified by a general intellectual deficiency or a lack of educational opportunities. Dyslexia is related with phonological processing deficits and slow, laborious reading [171] Poor performance in a range of “cerebellar” motor tasks, such as balance, quick pointing, peg shifting, and eye movement control are some of the traits of dyslexic personnel [171]. These observations, in conjunction with the absence of fluent, instinctive reading, led to the hypothesis that dyslexia includes cerebellar impairment as its primary neurobiological cause. Cerebellar dysfunction leading to a procedural learning deficit may be a feasible explanation for dyslexic readers’ poor reading skill acquisition, as dyslexia is classified as a distinctive disability connected to learning [171]. NDDs frequently co-occur, which highlights the likelihood of shared or overlapped biological processes in affected individuals, Figure 6a [171].

### 2.7. Strokes and Brain Tumor

Brain ischemia, also known as cerebral ischemia, is a disorder that develops when the brain doesn’t receive enough blood to support metabolic requirement [175]. As a result, the brain suffers from cerebral hypoxia or reduced oxygen supply, which causes cerebral infarction or ischemic stroke and the destruction of brain tissue [176]. The blood-brain barrier’s (BBB) integrity must be preserved for the central nervous system to remain in a state of equilibrium. In the neurovascular unit, which structurally makes up the BBB, brain endothelial cells interact with pericytes, astrocytes, neurons, microglia, and perivascular macrophages [175]. A severe neuroinflammatory response is triggered by brain ischemia to clear the injured tissue and get the brain ready for repair [68,176], but the severe neuroinflammation that takes place during the acute stage of a stroke is linked to BBB disruption, neural damage, and poorer neurological results (Figure 6b). Most laboratory-developed treatment approaches have focused on protecting neurons against the key pathogenic pathways that cause ischemia injury, such as excitotoxicity, oxidative stress, inflammation, or apoptosis [68,175,176].

The most common and fatal primary adult central nervous system malignancy is glioblastoma multiforme (GBM) [68,177,178]. GBM cells operate as reservoirs for recurrence and enter the brain parenchyma in a variety of ways, including as single cells [177]. Numerous transcriptional subtypes reflecting variable tumor genetics and epigenetics have been found because of the extensive molecular profiling of GBM [179,180,181]. The extracellular matrix, stromal cells, and tumor cells interact intricately with one another to create a dynamic and immunosuppressive GBM tumor environment that is highly resistant to current therapies [182].

Before being identified, malignant cancer tumour cells can infiltrate the circulatory system and distribute itself through blood medium until they find new host to develop [182,183]. The presence of cancer stem cells significantly facilitates the spread of cancer to various organs, including the brain. A small percentage of cancer cells are only capable of overcoming barriers, infiltrating neighbouring organs and tissues, and thriving there [177,184]. Cancer stem cells have the capacity to perpetually renew themselves and develop into more proliferative cells that make up malignancies [184,185]. The tumor microenvironment contains a number of elements that encourage the spread of cancer to nearby tissues or organs (Figure 6c). As cancer cells spread into tissues, their enzymes break down the proteins in the area, removing any barriers to the cells’ invasion [186]. It has been shown that tumor cells have increased the expression of serine and cysteine proteases and metalloproteinases, which targets the collagen-targeting elements of the ECM [185,186]. Lysyl oxidase, a collagen cross-linking enzyme, and collagen are increased in gliomas to encourage tumor cell motility and proliferation [183]. The patient mortality is significantly impacted by this cancer vasculogenesis [184]. Regions of the brain that are heavily vascularized promote the ongoing survival and functionality of cells [183,187,188]. Depending on the extent of the tumour, stromal cells, macrophages, astrocytes, endothelial cells, and microglia participate in microvascular proliferation in gliomas [126,184,188]. The perivascular niche (PVN), which is made up of stromal cells, is a crucial component that preserves and disperses the local stem cells. These cell types include adipocytes, neurovascular endothelial cells, pericytes, astrocytes, leukocytes, and macrophages as well as cancer-associated fibroblasts [184]. The development, migration, and metastasis of tumor cells are aided by the recruitment of stromal cells to tumor locations. In order to accomplish this, they release soluble cues such as growth factors, cytokines, chemokines, and other signalling entities like exosomes [184,185]. Tumor cells then interact with stromal cells to influence their microenvironment. There has been minimal improvement in the prognosis for people with Glioblastoma in spite of enormous efforts over the previous few decades [189]. Standard-of-care Glioblastoma treatment includes adjuvant temozolomide, concomitant chemoradiation with the oral methylator, and maximum safe surgical resection [190]. The extensive infiltration of tumor cells into the brain and the need to protect vital brain functions prevented complete surgical excision utilizing hemicraniectomies from being successful in curing the condition [189,190]. Its ubiquitous recurrence and poor prognosis are caused by an immunosuppressive and extremely diverse tumor microenvironment, limiting delivery through the BBB of chemotherapy/immunotherapy [189,190].

## 3. Advancements in Diagnostics Approaches

### 3.1. Electroencephalogram (EEG)

The electroencephalogram (EEG) is a recording of brain electric signals fluctuations recorded with the help of electrodes fixed on humans [191]. On the scalp, passive electrodes (Ag/AgCl) and, occasionally, sponge and flexible electrodes are used to conduct non-invasive EEGs. The measurement represents a broad portion of the cortex’s post-synaptic cell potentials on average within 6 cm^2^ [192]. The electrodes are also inserted within the skull to record EEGs, according to the size and electrode characteristics, which can offer precise measurements of cortical dynamics and electrical signalling [193,194]. The brain’s electrical information is passed through the electrodes to a device that monitors and records the data [4,195]. The human brain has numerous neurons. When neurons connect with one another, electrical impulses are produced. For instance, while sleeping and waking up, the neurons are active [195,196]. Using synapses, the neurons are closely linked [4,191]. The dense network of neurons serves as a conduit for suppressive or excitatory activity. The neural connections transmit information among neurons in the brain. The brain waves have an amplitude of around 10 mV and operate at very low voltage. In accordance with the activity of neurons in the brain, the voltage gets changed and gets recorded by the detector [4,196,197,198]. Data from recordings are then filtered and amplified. The output displays the voltage or digital values that have been converted and vary in accordance with the brain activity [191,192].

According to signal frequency, there are four primary types of brainwaves: infra, gamma, beta, alpha, theta, and delta. Infra waves have an extremely low frequency (<0.5 Hz) [199,200]. The speed of these waves is sluggish. It is challenging to find these waves. In contrast, gamma waves have a high frequency. In the brain, gamma waves are active while information is being conveyed [201,202]. These waves manifest when we are in a sympathetic or compassionate mood because they are rapid (<50 Hz). Delta brain waves are also sluggish ones. These things happen when we are relaxing or napping. These have an extremely low range and are hard to detect. The majority of delta activity is observed in new-borns. In elderly people, deep sleep phases are related to delta waves [203,204]. Patients with partial seizures, which cause short, unexpected gaps in attention, have shown evidence of delta waves interictally (between episodes). Low-frequency, high-amplitude waves with a frequency of 4 Hz or less define delta waves. Delta oscillations can exist while awake; they respond to opening the eyes and may even be strengthened by breathlessness [199,200,202,204]. Theta waves occur when the brain is in a relaxed state. Deep states of meditation also allow for the detection of these waves with frequencies of 8 to 12 Hz [199,200]. The alpha waves, like theta waves (8 to 12 Hz), are dominant when humans are in a thinking state or deep thoughts. These waves may also be present when one is meditating [200]. The alpha waves manifest in the states of attentiveness, learning, and tranquillity [61]. Beta waves are the desirable ones to be captured. These waves are in a state of awareness [202]. These waves are classified into Beta 1, Beta 2, and Beta 3 [71]. The Beta 1 operates between 12 and 15 Hz. Beta 2 is between 15 and 23 Hz. Beta 3 is between 23 and 37 Hz [202]. The most direct correlation between beta waves and consciousness or an alert, attentive, and aware state is consciousness [205]. Low-amplitude beta waves are related to active focus as well as to being active or agitated [100]. Motor choices are also correlated with beta waves (inhibition of movement and sensory response). In general, for visual stimuli, the brain’s occipital cortex reacts. For concentrating, the parietal region of the brain reacts [100,206]. This part becomes active when thoughts are deep. The brain’s temporal cortex region responds by producing speech. The human brain’s frontal region reacts in cognitive control [71,201].

**Figure 7:**
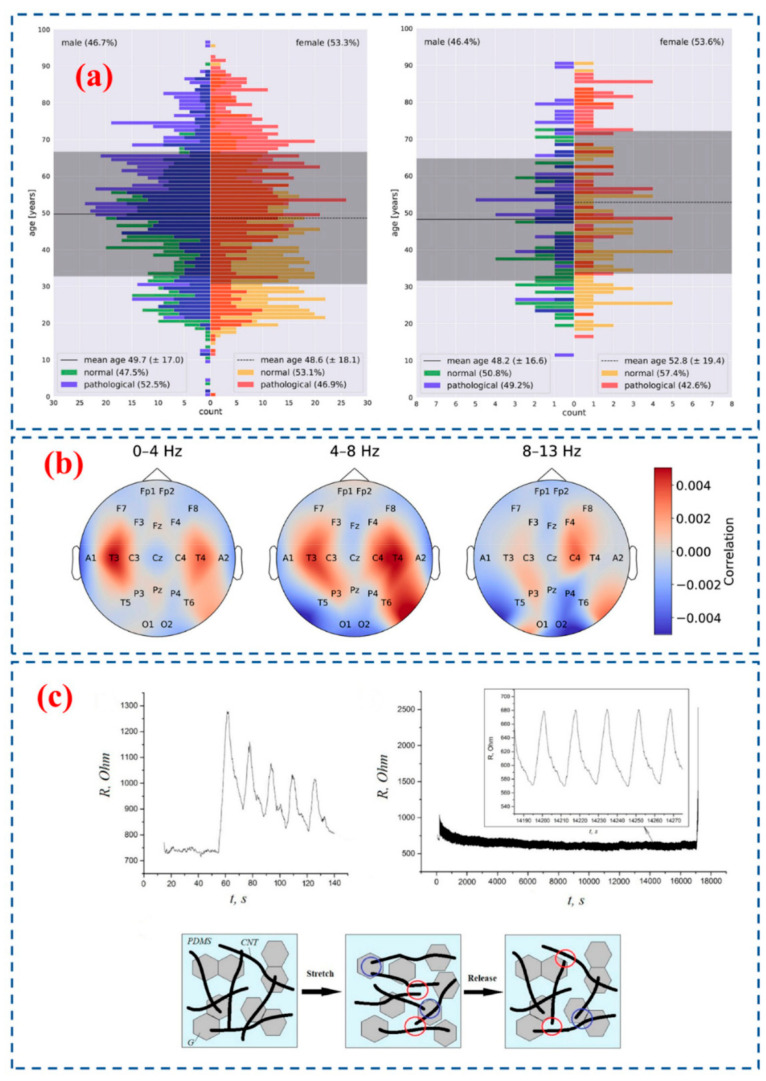
(**a**) The construction of the EEG histogram and the age pyramid with sections for male and female patients using ML (adapted with permission from [197] Elsevier, 2020) (**b**) The temporal electrode sites T3 and T4 exhibiting a pathological class with increase in amplitudes measured by neural networks (adapted with permission from [197] Elsevier, 2020) (**c**) Mechanism of conductivity of the hybrid PDMS/CNT biocompatible composites and their resistance vs. time plots (adapted with permission from [198], MDPI, 2021).

Computational neuroscience is an emerging field where quantitative EEG is used to study the electrical activity in the brain [201]. The brainwaves of people with the same traits, such as a similar age and gender range, are compared with the brainwaves of those who do not have brain disease using machine learning [206]. Through regression and classification ML models, previously, researchers have produced discrete and continuous data with supervised learning to develop a prediction model [100,205]. Using only data input that has been subjected to clustering and dimension reduction, researchers have produced discrete and continuous data with unsupervised learning to generate a prediction model [203]. ML for EEG analysis may be roughly divided into two categories: feature-based and end-to-end techniques [205]. Raw or hardly pre-processed data is accepted as inputs by end-to-end decoding techniques. Gemein et al. and co-workers used feature-based decoding to leverage a huge collection of features with end-to-end decoding using deep artificial neural networks (ANN) [197]. They created a complete analysis using about 3000 datasets with a minimum sampling length of 15 min from the Temple University Hospital (TUH) Abnormal EEG Corpus. They further employed Brain decode (BD), a formerly created and assessed deep learning toolkit for EEG. Figure 7a shows the data from Temple University Hospital, Abnormal EEG Corpus growth subgroups (left) and final assessment subgroups (right). A male and female patient-specific chronological pyramid was used to build the histogram. The pathological and non-pathological EEG recordings are distinguished by different colour coding [197]. From Figure 7b, increased activation at temporal electrode sites (T3, T4) is a marker of disease, particularly in low frequency ranges (0–4 Hz and 4–8 Hz) calculated using deep neural networks. At the occipital electrode sites, there is a strong negative connection with a degenerative category in the alpha frequency range (O1, O2) [197].

One of the simplest methods for gathering neural data from the human brain is dry contact electrode-based EEG collection, which offers various benefits, such as quick setup and improved wearability [207,208]. However, high contact resistance brought on by inadequate electrical interaction at the electrode-scalp interface continues to be a serious problem [208,209]. Nanostructures are recently used in MEMS and biosensors linked with EEG, which can affect synaptic transmission of associated neuronal networks [210]. Low-dimensional structures such as nanowires, nanospheres, nanotubes silicon/polymer structures, polymer-metal composites, and carbon materials are used in MEMS arrays. A study by Chen et al. describes stretchable polymer-based electrodes that may be safely utilized to monitor EEG data [71]. Since there is an optimal balance between mechanical and electrical characteristics in electrodes with a carbon content of around 45%, brain signals have been detected using these electrodes. The polymer electrode is a viable replacement for traditional gel electrodes [61]. Biologically compatible carbon nanotube hybrid composites that are flexible and extremely conductive are explored by Barshutina et al. In their study, a safe wearable electronic electrode with CNT hybrids as active material was used, as shown in Figure 7c, which leads to new spectrum of opportunities for carbon nanostructures as biomaterials [198]. CNT/PDMS and silicone-based biomaterials are also used as electrodes [211]. Carbon-based materials and metal–organic structures can be made biocompatible via chemical and biological functionalization [212,213]. Chemically functionalized carbon nanotubes have a lower toxicity, but since they are thought to be non-biodegradable, they are still viewed with skepticism [207,214]. Lately, it was proven that oxidative enzymes may break down functionalized CNTs and make them biocompatible [212]. This discovery is providing a fresh viewpoint for the application of CNTs in medicine [213,215].

### 3.2. Computed Tomography Scan (CT Scan)

CT scans are mostly used to identify a brain stroke. CT scans can offer the necessary data for carrying out the emergency operations immediately following a stroke [216]. A CT is more resistant to noise and somewhat less costly [207]. Compared to other imaging methods like MRI, a CT is quicker and more easily accessible to patients. The initial radiological test carried out on the patient after a stroke is a non-enhanced CT [217]. The existence of an ischemic lesion is indicated by a hypodense structure in the CT images. However, a CT scan makes problematic lesions difficult to see [208]. Additionally, it encounters difficulties while trying to find tiny infarcts in the brain [218].

It is common practice to utilize CT scanning of the head to identify infarction (stroke), tumours, haemorrhage, and nerve damage [219]. Of the conditions, hypodense (dark) structures can signify edema and infarction; hyperdense (bright) structures correspond to haemorrhage and trauma. Tumours can be identified by the edema that surrounds them or by the inflammation and structural distortion they induce [70,218]. Three major advancements in the instrument of the CT were established in the past decade: (i) Electron Beam Tomography (EBT), (ii) Dual-source CT, and (iii) CT perfusion imaging [212]. Electron beam tomography (EBT) has a major advantage of scan speeds and clear imaging of moving structures [217]. EBT is one kind of CT in which a large X-ray tube is designed and built so that only the path of the electrons, travelling between the anode and cathode of the X-ray tube, are spun using deflection coils [216]. Contrary to conventional single tube systems, dual source CT scanners use two X-ray tube detector systems that are mounted on a single gantry at 90° in the same plane [216]. Dual source CT scanners enable quick scanning with higher time resolution by acquiring a full CT slice in just half a rotation [216]. Fast imaging lessens motion blurring at high heart rates and may shorten the time needed to hold your breath. A contrast agent is injected during a special type of CT called “CT perfusion imaging” to measure blood flow via blood vessels [216,218].

Deep learning and artificial intelligence are mostly used to recognize strokes accurately and automatically [212]. These findings do not function as a stand-alone component of the detection procedure [216]. Computer-aided approaches can be useful to help doctors in the stroke detection procedure since it is a very sensitive treatment process. Metallic nanoparticles, especially gold (AuNPs) with distinctive biochemical features, have been employed in sensors and CT detectors [220]. The most significant applications for AuNPs are those that result from their exceptional surface characteristics [221]. In order for AuNPs to be stable, dispersive, and soluble, their surface must be functionalized [222,223]. The coupling of NPs with bio-macromolecules is the most incredible example of surface functionalization and chemical modification [222]. Prostate-specific membrane antigen (PSMA) RNA aptamer-modified AuNPs were used in one investigation to target prostate cancer cells alone. Due to their selective adsorption and contrasting characteristics, the proposed PSMA aptamer conjugated AuNPs displayed a higher CT signal for a specific tumor [224]. Additionally, Li et al. created fluorescent AS1411 aptamers that were targeted for nucleolin. The (AS1411-DA-AuNPs) conjugates displayed substantial X-ray attenuation, high hydrophilicity, and outstanding biocompatibility [225,226].

### 3.3. Angiogram

A catheter, a long, flexible tube, is usually introduced into a vein in the arm or leg during cerebral angiography [227]. A doctor inserts a catheter into the patient’s vein and introduces a particular dye into the bloodstream arteries leading to the brain [228]. It is a technique for creating x-ray images of the interior of blood arteries [222]. The standard for determining intracranial circulatory (IC) issues is cerebral angiography [229]. The IC rises in the event of brain death, obstructing blood flow in the internal artery and vertebral arteries. Angiography will reveal any intracranial haemorrhage filling when these arteries enter the skull. In addition to necrosis brought on by brain damage, the destruction of the intracoronary vascular space also plays a role in the lack of intracranial flow [227]. The usual cerebral circulatory blood, in which the low resistant intracranial arteries fill preceding the higher resistance vascular arteries, is reversed after cerebral cell death. External circulation is still there after brain death and fills quickly [230]. Certain individuals may have a cessation of supratentorial circulation, yet prolonged posterior ventricle blood flow may still be present, possibly because of the cerebellum tentorium’s supportive role against increasing cortical tension [229]. Angiogram is intrusive, time-consuming, and relies on the accessibility and expertise of the technician despite the benefits of precision and resolution. Furthermore, angiography may unintentionally restrict blood flow in the leftover veins and harm a patient’s transplantable tissues [227,228,231].

Zhang et al. used a deep learning algorithm based on U-nets that combines pre-processing alongside tracking and segmenting brain blood arteries in digital subtraction angiography (DSA) images. Using precision, responsiveness, and selectivity, Zhang et al. contrasted the outcomes of the deep learning technique with manually labelled truth sets [232]. Their findings revealed that the suggested method had an accuracy of 97% [232].

### 3.4. Positron Emission Tomography (PET)

Brain metabolic activity has typically been the main focus of brain PET investigations [233,234]. It is well known that the performance and functionality of synapses are tightly correlated with a PET signal, which represents both aerobic glycolysis in microglia and oxidation metabolism in neurons [235,236]. Because a variety of neuropsychiatric disorder processes—including altered cellular signalling pathways and mitochondrial bioenergetics, reduced dopamine release, a build-up of cytotoxic peptide species, and disordered electric signals—can cause synaptic failure. When measuring cerebral activity and the spread of foreign agents throughout the head, PET analyses emissions from substances that have been put into the circulation and are highly active [237,238,239]. Computer-processed multi-dimensional pictures of the location of the substances throughout the brain are obtained using the electron emission data. The efficacy of PET detectors in comparison underwent a significant step-change with the development of solid–state silicon photomultiplier (SiPM) detectors. The size of the detector in the longitudinal axis has been growing as a recent direction in improving PET physical functioning to boost scanner responsiveness [237,240]. Although this is by no means a simple procedure, it is a rather evident development; yet end users may experience practical difficulties when installing these bigger scanners [235,241].

**Figure 8:**
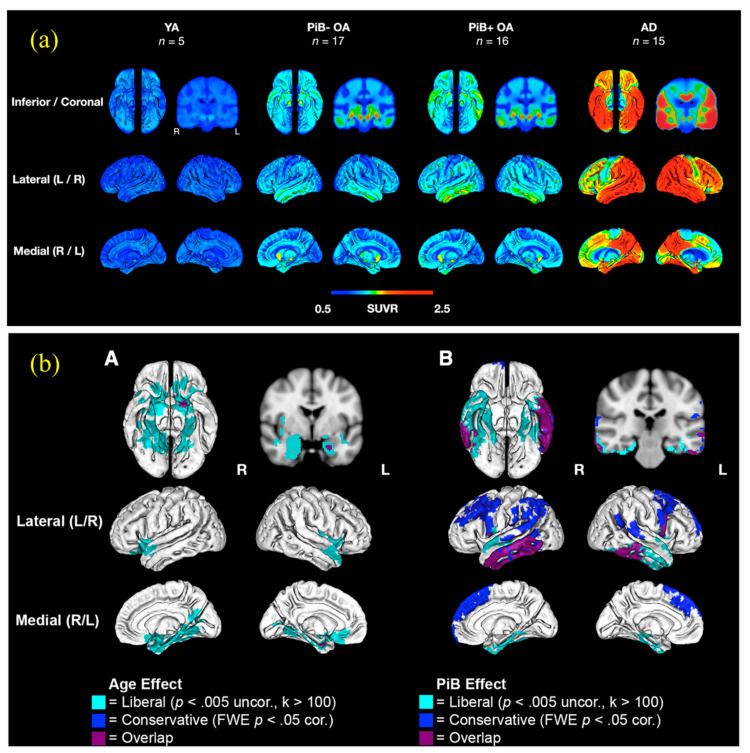
(**a**,**b**) in vivo PET spatial range of AV-1451 retention correlated with age, Aβ, and tau. (Adapted with permission from [233], Cell Press, 2016).

Recently, as shown in Figure 8a,b, the authors investigated retention rates in cognitively healthy older individuals in comparison to young controls and AD patients using the tau PET agent 18F-AV-1451 [233]. In health and longevity, tau tracer uptake was variably correlated with age and amyloid (as determined by PiB PET). Greater tracer retention in areas of the medial temporal cortex was associated with older age and indicated poorer episodic memory ability [223]. Global cognitive reduction was linked to PET identification of tau in additional subcortical areas, which required the presence of cortical amyloid. Additionally, trends of tracer persistence matched the Braak stage of fibrillary tau disease rather well. In this study, patterns of tau tracer retention in relation to age, cognition, and amyloid deposition were established in normal aging [233].

Future interest in image acquisition research has been sparked by the recent finding of 3D image capture in PET/CT. In contrast to 2D mode, operating in 3D (dimensional) configuration is a recent development in PET imaging that increases pulse sensitivity by a factor of 4–6 [235]. The disadvantage of 3D acquisition is an enhancement in the scattering proportion from 10–15% to 30–40% in the number of dispersed occurrences that are identified as a percentage of all coincident occurrences [242]. Standard medicine PET/CT scanners may now use Time of Flight (TOF) imaging due to the picosecond timing accuracy rendered feasible by rapid synchronization circuits and the quick fading of detectors. Even though the idea for TOF-PET had originally been put forward years earlier, it had only just begun to be implemented in research PET detectors [242,243]. Hybrid PET/MR (Magnetic Resonance) for clinical usage are available that can capture MR and PET imaging data concurrently without significantly affecting each technology’s usefulness or efficiency [239]. Due to the cross of the PET and Magnetic Resonance MR signals, simultaneously collecting MR and PET data involves considerable technical hurdles. Analog photomultiplier devices are supplanted by high-speed, magnetic field-insensitive annihilation photodiode detectors (APDs) to reduce this interference in the signalling [242,243].

### 3.5. Magnetic Resonance Imaging (MRI)

In MRI, a specific pulse sequencing and magnetic force gradient generate a develop a specific tissue signal and contrasts to identify various bodily tissues [244,245]. The T1 and T2 relaxation principles are the same throughout all MRI sequences, although variable pulse sequences are used. Most illnesses produce an increase in the hydroxyl content of the infected tissue, which causes them to stretch more slowly than healthy tissue and provide a distinct response and imaging look [246,247]. The most used cerebrospinal fluid sensitive (CSF) sequence procedure is two-dimensional phase contrast imaging with speed labelling. A typical signal is received from flowing CSF, but no signal is generated from stationary CSF when two RF pulses are administered in opposing directions and balance each other out. This sort of sequencing is mostly used to evaluate CSF anomalies [244].

The evaluation of several nanoparticles with distinctive optical and magnetic characteristics that can offer real-time tracking is a recent advancement in MRI [248]. Nanoparticles (Fe, Au, Cu, Ni) have also been utilized to create photoacoustic imaging, another kind of nanoscale medical imaging, in addition to conventional fluorescence imaging and MRI [244,249,250]. The method employs an electro-optical wave that is created by the thermal cell expansions brought on by a laser pulse. The single nanoparticle imaging of tissues has been done using the resultant ultrasonic emission [250]. Although other plasmonic nanoparticles have been used with success, gold nanoparticles are particularly well suited as high contrast agents for photoacoustic imaging [251,252,253]. In various types of neurosurgeries, functional MR scans are frequently utilized for brain mapping both before and after surgery. Quicker scans are now feasible because of the improved MRI software, enabling the previously impractical imaging of the neurons due to motion artifacts [244,249,250].

Patients may find the noises from the MRI scanners to be quite upsetting. In order to increase patient comfort, manufacturers are producing an improved scanner with less stress and sound intensity [253]. Additionally, there are now scanners on the market that offer an acoustic experience with lighting, sound, and films to aid with patients’ phobia and produce a scan that is motionless [252,253]. As scanner sizes and operating power requirements shrink, they may be deployed with reduced operating costs. There are many opportunities for MRI methods to advance, and this research and has a bright future [244,245,246].

### 3.6. Mass Spectrometer and Chromatography

Liquid chromatography tandem mass spectrometry (LC-MS/MS) is a chemical analytical technique that allows researchers to concurrently quantify the quantities of various hormones as well as the activity of their synthetic enzymes [254]. Conventional biochemical studies employ precursors that are tritium labelled, which is risky, expensive, and frequently requires repurification before use, which is a time-consuming process. Furthermore, the nature of some steroids, such as 11-hydroxytestosterone (11-HT, a powerful androgen in fishes), precludes the use of a tritium label [15]. This constraint prevents a thorough biochemical analysis of this conversion process. LC-MS/MS has been used in a growing number of field and laboratory research for a diverse range of reasons. It is more sensitive than conventional antibody-based approaches [15]; it makes use of automated procedures to cut down on sample preparation time and test kit costs; it is highly selective for specified metabolites and has negligible effective reactivity with hormones that are closely related; it is adaptable to a variety of sample kinds; and it enables the simultaneous assessment of several hormones along with synthetic enzymes from a single sample without the need to utilize various time-consuming androgen and estrogen separation techniques, such as thin-layer chromatography [254]. Furthermore, it enables scientists to identify the biochemical conversion of any precursor molecule, many of which may be obtained at a minimal cost. By allowing for the identification of novel biomolecules in samples, LC-MS/MS is a technology that is perfect for researching the evolution of hormones and behaviour in animal models that are non-traditional [15].

More than 10,000 proteins, representing more than 60% of the expressed proteome, were discovered in recent research that involved the optimization of the LC/LC-MS/MS technology to analyse a human brain specimen of Alzheimer’s disease [255]. Contemporarily, after administering acute methamphetamine to rat pups, the novel approach that combines lyophilization with LC- electrospray ionization-MS/MS was tested on actual samples taken from the nucleus accumbens. It was demonstrated that the assay could be used to analyse 3-methoxytyramine, dopamine, 3,4-dihydroxyphenylacetic acid, and homovanillic acid, substrates simultaneously in micro dialysis samples taken from rat brains and to track any minute changes in their concentrations over time after exposure to methamphetamine [255].

### 3.7. Functional Near-Infrared Spectroscopy

The Functional Near-Infrared Spectroscopy (fNIRS) technique is a non-invasive, non-ionizing method of measuring and visualizing the functional hemodynamic response to brain activity [256]. The fNIRS provides a glimpse into the brain based on blood oxygenation without the need for a large, stationary scanner [248,251]. This optical imaging method looks for changes in the absorption of near-infrared light, which typically has wavelengths between 750 and 1200 nanometres [256,257,258]. The amounts of oxygenated, deoxygenated, and total haemoglobin within the brain can be spectroscopically examined using near-infrared light, which can pass through the scalp and skull for several centimetres [252,259]. Variations in the amount of diffuse light that reaches the detector can be used to determine changes in cerebral haemoglobin concentrations by beaming near-infrared light on the scalp and positioning a detector a few centimetres away [258]. The fNIRS offers a proximate indicator of regional brain activity analogous to fMRI. Despite having a lower spatial resolution than fMRI, fNIRS has a significant advantage where it allows the individuals to move around freely, speaking and interacting with their surroundings while being scanned [248,251]. Similar to EEG electrodes, the light sources and detectors of the fNIRS can be installed on a wearable cap to provide a portable, lightweight, and affordable array that can be worn in range of settings [251]. While EEG monitors the rapid, electrical responses associated with neural activity, the fNIRS depends on neurovascular coupling and assesses the hemodynamic response [258,260]. The fNIRS is especially well suited for populations and investigations where other imaging modalities are insufficient, such as those involving infants and children; activities requiring movement and interaction, and clinical settings [251,259]. The most prevalent fNIRS application fields include behavioural and cognitive neurodevelopment, perception and cognition, psychiatric illnesses, neurological applications such as brain injury, neurological diseases, and epilepsy, and stroke [255,256,257,260].

## 4. Advancements in Treatment Approaches

### 4.1. Use of Nanotechnology

Gold, silver, iron, lipid-based, dendrimer, and polymeric nanomaterials make up the majority of the nanoparticles used in brain tumor treatment [261]. A new age in brain imaging may have begun thanks to multi-functional nanostructures, which are typically described as having three exterior dimensions at a nanoscale no larger than 100 nm in diameter [262,263]. These interactive nanoparticles allow for the cellular and molecular monitoring of tumor progression [264]. Tumor diagnosis has improved in sensitivity and accuracy thanks to nano diagnostics, including MRI using dendrimers and metallic nanoparticles [263,264]. Early detection is essential for increasing survival rates since smaller tumours may be treated more effectively, which is now possible with nanoparticles. This section describes the various use of nanoparticles and nanotechnology for the treatment of brain diseases [265,266].

#### 4.1.1. Drug Delivery

Drug delivery nano systems are regarded as cutting-edge technology platforms that may deliver bioactive compounds to specific tissues while adjusting their physicochemical characteristics to improve their bioavailability and solubility [69,267]. Neurobiological barriers like the blood-cerebrospinal fluid barrier (BCSFB) and the blood brain barrier (BBB) prevent some therapeutic drugs from reaching the CNS and reduce their treatment effectiveness. In this sense, the nanoparticle is seen as a potentially effective tactic to enhance medication tailoring to the brain and boost bioavailability [263,265,266]. The use of nanotechnology in medical research, the creation of novel treatments, and the enhancement of treatment effectiveness are all receiving a lot of attention. For antigen-specific signal transduction pathways, antigen-loaded nanoparticles offer a number of benefits, including prolonged antigen release, the targeted delivery of antigens and aptamers simultaneously, the creation of antigen warehouses at the site of injection, the appropriate production of B-cell autoantigens, and a boost in the absorption and stimulation of immune responses against stromal pathogens [268,269,270]. These nanoparticles are joined with certain epitopes and antigens associated to the autoimmune response in MS. These nanoparticles can stimulate Treg cell and dendritic cell development, control T-cell function, and restore immunological tolerance. Nano drug carriers also play a significant role in penetrating the BBB, namely liposomes, micelles, dendrimers, and polymeric nanostructures [261,271].

Lipid vesicles such as liposomes, solid lipid nanoparticles (SLN), nanostructured lipid carriers (NLC), and microemulsions are thought to be the best means of delivering drugs to treat brain diseases [272,273]. They have the capacity to penetrate the brain’s capillaries, vascular endothelium, and the BBB, lessening their negative effects on nearby tissues [274,275]. Additionally, by appropriately decorating their surfaces, lipid nanocarriers may be created to engage with biomolecules or cellular receptors found in the BBB, even delivering medications that usually cannot penetrate the BBB [2,276,277]. NLCs regulate the amounts of the liposomes to maintain their stable state at body and ambient temperatures. By substituting a lipid membrane that is rigid at both room and skin temperature for liquid lipids in oil-in-water (O/W) nano emulsion, SLN are colloidal carriers with greater physiological stability [11,275,278]. The lipid core of SLN is often stabilized by stabilizers and composed of fatty acids, diacylglycerols, glyceryl, esters, paraffin, or steroids [277]. Stearic acid, stearyl alcohol, glycerine monostearate, and other common solid lipids utilized in NLC formulation include sesame, olive, jojoba, soybean, and peanut oil. Natural polymers like collagen, starch, and cellulose are effectively used as nano micelles [2,277]. The natural biopolymers’ drawbacks, such as poor solubility in solvents and may be readily chemically changed with side chains thanks to the existence of multiple functionalities change with different functional groups [2,277]. The latest developments in bioengineering have made it possible to create novel amphoteric hydrophilic polymers made of polysaccharides or proteins that are employed for specialized applications, such micellar carrier drug delivery [248,279]. The self-assembly process has a significant impact on the micellar sizes and polydispersity [270,276].

Highly branched, monodispersed, chiral polymeric biomolecules known as dendrimers have a lot of surface functional groups [280]. Dendrimers comprise three separate architectural elements: an internal core, an inside layer, and an outer layer [269]. The surfaces have a range of reactive terminals that enable multifunctionality and tightly packed border to increase the ability to load the medicine, and the interior holes are appropriate for doing so [69]. A polyamidoamine (PAMAM) dendrimer loaded with Au nanoparticles has been used for the detection of Alzheimer’s disease. PAMAM with Au was also used to rapidly detect tau protein and DNA protein binder-43 simultaneously in the brain in another study [64,69,267]. A possible method for the early identification of brain illnesses might be the quick identification of these two proteins, which are known to be hallmarks of neurological diseases [281,282,283,284]. Dendrimer-nanoparticle hybrids may be investigated for multiple detection and targeted delivery to the brain as well as brain disease diagnostics [64].

**Figure 9:**
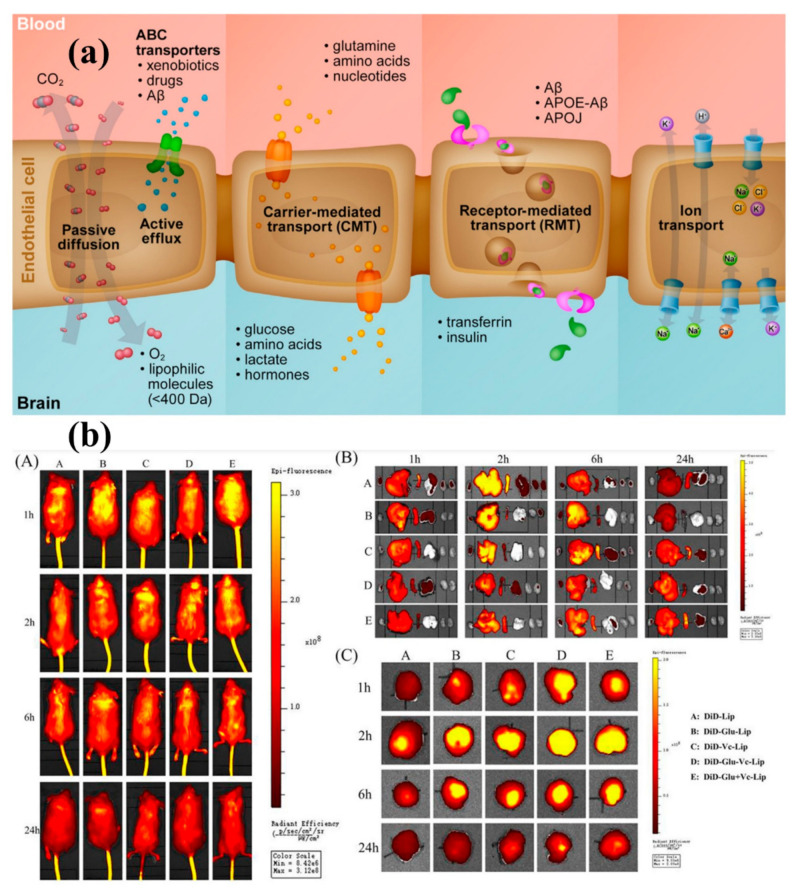
(**a**) Transport mechanism of O_2_, CO_2_ and lipophilic molecules across BBB (adapted with permission from [72] Elsevier, 2020) (**b**) In vivo and ex vivo images of drug loaded liposome administrated mice after 1, 2, 6 and 24 h (adapted with permission from [5], Elsevier, 2018).

For effective BBB crossover, the transport mechanism of targeted therapies to the CNS, and their role in the therapy of neurodegenerative illnesses like multiple sclerosis, a variety of polymers and their corresponding nanoparticles were investigated (Figure 9a) [72]. Glucose, certain amino acids, and monocarboxylates can be taken up by protein transporters on the luminal endothelium and transported into the central nervous system (CNS), whereas those on the vascular endothelium side release glutamate and nucleotides. Receptor-mediated transcytosis (RMT) (Figure 9a) involves attachment to a receptor, endocytosis of the receptor combination within a vesicle, separation of the combination, and exocytosis of the ligands to move a variety of biomolecules across the BBB, including insulin and ferritin. Ion channels are also present, and they are in charge of exchanging ions across the BBB in order to preserve brain homeostasis [72].

It is possible to produce polymeric nanostructures utilizing a variety of synthetic and organic biopolymers and different preparation techniques. Additionally, their surface can be functionalized for targeted brain stimulation [285]. The polymers that are employed to make these nanostructures must be biodegradable and bio compatible [286]. The processable, biocompatible, and Food and Drug administration (FDA) approved polymeric poly(lactic-co-glycolic acid) (PLGA) nanoparticles are suitable drug carriers [287]. The ability to readily load PLGA nanoparticles with a range of compounds is one of the main benefits of employing them [288]. Attempting to control the PLGA’s physicochemical characteristics, such as the lactide-to-glycolide ratio, crystal parameters, molecular weight, thermal properties, and surface coating makes it simple to observe the reaction mechanism of nanoparticle degradation and the rate at which the encapsulated molecules release the drugs, which opens up whole new spectrum of opportunities [288]. Polylactic acid, polycaprolactone, polyurethane, polysiloxane, polysaccharides, polyethylene glycol, and poly-ethylene vinyl acetate have been recently used as carriers for drug delivery [69,289].

Following the systemic treatment of drug-loaded liposomes, animals carrying cerebral C6 gliomas were depicted in in vivo pictures in Figure 9(bA) at 1, 2, 6, and 24 h [12,288]. After that, the mice were killed at pre-determined intervals, and the organs that could be scanned included the brains, hearts, spleens, livers, lung, and kidneys [5]. Figure 9(bC) displays ex vivo pictures of the brains, whereas Figure 9(bB) displays pictures of other organs. The study’s findings were supported by the brightest fluorescence intensity of all the liposomes in the brain, which was plainly seen 2 h after injection [5].

#### 4.1.2. Biomarker Detection

A biomarker is an identifiable chemical or signal that is closely related to one particular ailment or state [290,291]. The ability of the biomarker to distinguish between normal and unwell people, as well as its specificity in describing the disease stage, are crucial for disease treatment [291,292]. Different biomarkers have been found in brain illnesses and diseases, but the absence of appropriate procedures makes it difficult to use them [16,293]. NPs have proven to be extremely effective at identifying critical markers of brain illnesses and diseases [294,295]. Plasma levels of ubiquitin-C-terminal hydrolase-L1 (UCH-L1) have been shown to be considerably higher in patients with traumatic brain injury (TBI) compared to healthy people, suggesting that it may be used as a biomarker for the disease [290,293]. An innovative technique using surface plasmon resonance of Au NPs may quickly and successfully identify the biomarker UCH-L1 in TBI patients with 100% sensitiveness and specificity, according to recent research [296,297,298]. Additionally, there is a strong correlation between A level and dementia and other linked disorders [299,300]. According to studies, customized magnetic nanoparticles may reliably and securely identify A plaques in the AD mouse model [295,301,302,303]. Additionally, it has been demonstrated that anti-cholesterol antibody-bound magnetic NPs are efficient in identifying high cholesterol levels, which is a crucial indicator of AD [304]. Additionally, according to a recent study, fluorescent NPs can be utilized to identify AD [290,292].

#### 4.1.3. Disease Specific Treatments

In mice models of TBI, it has been shown that encapsulating ROS-reactive compounds inside the NPs is beneficial in reducing neurological deficits [305,306]. According to a recent study, administering cerium oxide (CeO_2_) NPs to TBI rats can considerably lessen brain damage by improving antioxidant qualities and cognitive function [298,307]. According to studies, administering immunoregulatory NPs can significantly lessen inflammation and swelling as well as ameliorate motor deficits in TBI animal models [306,308].

The accumulation of Tau protein and Aβ is responsible for the occurrence of Alzheimer (AD) and Parkinson diseases (PD) [309,310]. Sonawane et al. used cadmium sulfide nanoparticles encapsulated with protein, which effectively restricts the fibrillization of Tau protein growth rates by 63% [311]. A serine regulator known as GSK-3 has been linked to the development of Aβ, which is responsible for phosphorylation of Tau proteins, and the progression of AD [310]. Additional research suggests that GSK-3 controls nerve cells in conjunction with histamine deacetylase (HDA) proteins [312,313,314]. Likewise, it has been shown that GSK-3 and HDAC inhibitors are successful at reducing AD. Au NPs have also been found to promote anti-inflammatory activities and enhance antioxidant capacity to elicit neuroprotective benefits in an AD rat model [315,316]. Additionally, it has been demonstrated that surface coated Au NPs can decrease Aβ accumulation, with the impact varied depending on the NPs’ dimension and surface characteristics [311,314,317]. Au NPs with negative surface potential have been shown to drastically lessen Aβ fibrillization and the accompanying cytotoxicity in an AD model [318,319]. Additionally, a recent study indicates that smaller Au NPs are more effective than bigger ones at inhibiting A fibrillization [320]. Polydopamine nanoparticles (NPs) loaded with metformin provide anti-inflammatory and antioxidant functions. On the other hand, a recent study [318,319] found that Se, iron, and chromium are some of the vital components that are noticeably enhanced in HDA patients’ blood when compared to those of healthy people [321].

Among the advancements, the following benefits of using nanotechnology in TBI therapy overall improved BBB protection, targeted medication delivery to wounded brain regions, enhanced cell membrane healing mechanisms, and nano-structured scaffolds for nerve tissue creation [309,310,322]. Currently, targeted medication delivery to the brain by different NPs in nanomedicine appears to be a viable option. NPs stand for medication delivery because to their unique properties of being biodegradable, biocompatible, more stable in blood, low immunosuppression, and non-toxic to the brain [320,321,322].

### 4.2. Wearable Sensors

Tremors are involuntary, oscillatory, rhythmic movement of any body part, which comes under general movement disorder. Rest tremors occur in a relaxed body part that is fully supported by gravity [323]. Action tremors, which include postural, isometric, and kinetic tremors, are tremors that are caused by the voluntary muscle contraction. At rest and when moving, everyone experiences high-frequency, low-amplitude physiologic tremors that are not symptomatic [324,325,326]. An essential tremor is the most typical pathologic tremor. The Parkinsonian tremor is far less common, usually asymmetrical, manifests at rest, and fades away with intentional movement [327,328]. A sudden onset, spontaneous remission, shifting tremor features, and extinction with distraction are traits that are consistent with psychogenic tremor [328]. The classification of the tremor based on its topographic distribution, triggering condition, and frequency is the first stage in the patient evaluation with tremor [324]. A complete history and physical examination provide clinical data used to make the diagnosis of tremor. Finding the underlying reason is crucial because prognosis and particular treatment options can vary significantly [323]. In terms of quantifying postural instability, the Pull Test is the most used clinical procedure [327]. For continual assessment of motor abnormalities in essential tremor and Parkinson’s disease, applications of wearable sensors or body-fixed sensors, particularly kinematic sensors, for long-term monitoring are being employed [329,330,331]. Wearable sensors and the IoT, two rapidly developing technical fields, appear to offer a sophisticated and ingenious method of providing pervasive healthcare services to the senior population, elevating healthcare facilities to a superior level of omnipresence [323,324,327]. Due to its superior solution-processable fabrication, chemical functionality, electrical conductivity, and mechanical durability, a growing number of nanostructured materials, including carbon nanotubes (CNTs), metal oxide nanoparticles, polysiloxane nanofibers, and graphene, have been used in flexible pressure and tensile sensors recently [332,333]. Additionally, electronic textiles with distinctive qualities like permeability, elastic modulus, and washability open up a wide range of possibilities for wearable technology. However, when used in particular settings, pressure and tension sensors have a number of drawbacks that prevent their widespread commercial application [334,335,336]. The sensitivity to minute strain changes in the human body is hampered by the low detection limit of many mature tension sensors now in use. It is necessary to address the pain caused by the interaction of electronic equipment with the human body and the incomprehensibility of wearable technology [333,334].

### 4.3. Bioprinting

**Figure 10:**
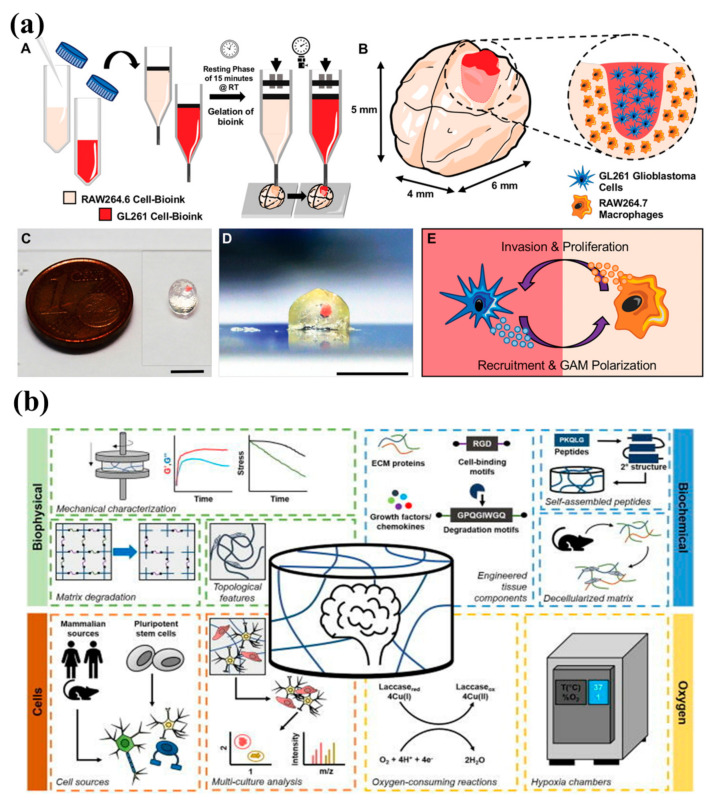
(**a**) Schematic representation of the bioprinting process and the bioprinted mini-brain (**aA**) Preparation and two-step bioprinting of the mini-brains. (**aB**) Close-up and cross-sectional view of the bioprinted mini brains. (**aC**) Glioblastoma area in red. (**aD**) Cross-section in the frontal plain (**aE**) Schematic of interaction between glioblastoma cells and macrophages (adapted with permission from [351] Wiley, 2019) (**b**) Components of engineered brain-mimetic platforms involving biophysical parameters, oxygen content, biochemical and cellular composition (adapted with permission from [352] AIP, 2021).

A lot of the drawbacks of existing modelling methods can be overcome by bioprinting technologies, which can create personalized 3-dimensional tissue models with good reproducibility, flexibility, and scalability [332]. Bio fabrication technologies can be divided into groups depending on whether biological components are added to constructions after the creation of devices or encased in biomaterials during the fabrication process [333,337]. In comparison to the cell-seeding strategy, the cell-encapsulating approach allows for better control of the quantity and location of deposition of cells and molecules, improving reproducibility [338,339]. In contrast to seeded cells, which only get ECM cues from the side that contacts the hydrogels, hydrogel-encased cells receive ECM signals from all directions, simulating their physiologic states [338,340,341]. While numerous processes, like fused deposition modelling, selective laser sintering, and electrospinning among others, are capable of producing devices or cellular scaffolds with high throughput and resolution, they are not frequently employed for cell encapsulation. In this regard, due to its capacity to accurately manipulate tissue architecture and matrix properties to encapsulate cells in biomaterials with high survivability, 3D bioprinting plays a major role [342,343]. The distribution of cells, active compounds, and biomaterials can be precisely controlled by bioprinting (Figure 10a). Additionally, this method creates in vitro tumor models with bionic structures and physiological systems that replicate the salient features of the tumor microenvironment [344]. These models can serve as reliable platforms for research on the development of tumours, their interactions with their environment, angiogenesis, motility, invasion, and intra- and extravasation. Additionally, high-throughput medication screening and validation as well as the prospect of individualized cancer therapy research can be done using bioprinted tumor models [345]. For several neural conditions, the therapy options are limited, and a few of them simply offer symptomatic alleviation. The substantial economic consequences of CNS disorders include not only the cost of care but also the lost productivity of patients and their care takers, who may incur a heavy financial, emotional, and practical burden in caring for family members who have permanent disability [326,346]. The medicines for fully restoring neural function, despite the emergence of many clinical treatments, are still in their infancy, which has prompted researchers to look to neural tissue engineering techniques for the repair of CNS illnesses. Potential therapeutic measures include implanting tissue engineering bioprinted scaffolds containing cells or drugs at the injury site of the CNS [347,348].

The primary components required for bioprinting are bioink, cells, and biochemical factors [348]. The bioink is typically a hydrogel that is biomaterial-based and offers mechanical and biological support [347]. Apart from the biological compatible, degradable, chemical, and mechanical characteristics including viscosity, elastic modulus, and compressive modulus are also vital bioink aspects that must be taken into account [344]. The bioink must meet the requirements for printable parameters in order to match the low mechanical stiffness associated with brain tissue and maintain the appropriate shape and structure when the material is placed. This presents a significant difficulty in matching the mechanical strength [349]. It is possible to replicate the high level of complexity present in brain tissue more precisely by combining multiple cell types [349].

In general, neural scaffolding should possess three essential qualities: neurocompatibility, which permits the adherence and expansion of neighbouring nerve cells; elastic properties, which can mimic the mechanical characteristics of native nerve tissues; and hierarchical microarchitecture, which exhibits biomimetic features as well as physiochemical characteristics of human neural tissue extracellular matrices (Figure 10b) [350].

#### Biomaterials Used in Bioprinting

Gelatin is a collagen hydrolysis product. Due to the intrinsic bioactive properties of gelatin and its derivatives, such as integrin binding Arg–Gly–Asp sequences and matrix metalloproteases digestion sites, they are frequently employed in 3D tissue modelling. In comparison to tumor cells or perivascular niche (PVN) cells cultivated alone, PVN cells and GBM cells cocultured in a 3D gelatin matrix showed increased angiogenesis and ECM remodelling [353]. Gelatin-based materials are frequently employed as bio inks in extrusion-based bioprinting because of their favorable rheological features and thermally sensitive traits. Gelatin hydrogel has been used to encapsulate hepatocytes, and the 3D-printed tissue has remained viable and functional after two months of culture [353]. In order to increase the gelatin’s printability in terms of a longer bioprinting window and higher resolution, it can also be mixed with synthetic materials, such as PU [354,355].

In the majority of bodily tissues, collagen is an integral part of the ECM. Although fibrillar collagen type I is essentially missing from the brain, collagen types IV and V are prevalent in the vascular basement membrane. Therefore, biomaterials produced from collagen are suitable for simulating the BBB [354]. The plethora of cell binding sites, well-studied pH- and temperature-based gelation mechanisms, and variable mechanical qualities to satisfy tissue-specific requirements have led to the use of collagen biomaterials in numerous glioblastoma investigations [354,355]. The accuracy of bioprinting can be increased by enhancing collagen concentration or adding riboflavin to the prepolymer solution. Riboflavin is added to collagen bioinks to boost their storage modulus, which enhances printability [356]. Collagen-based bioprinting has been employed in tissue engineering applications such as heart regeneration and liver modelling. Gelation of collagen-based bioinks is often thermally regulated or pH-driven [355].

Hyaluronan (HA) is a negatively charged, linear polysaccharide that is produced in the plasma membranes of neurons and glial cells. It is made up of alternating d-glucuronic acid and N-acetyl-d-glucosamine [95]. HA-based hydrogels are the most appropriate matrix materials for mimicking brain tissues and brain tumours due to its preponderance in the brain and glioblastoma stroma and its crucial function in controlling numerous physiological and pathological processes through interaction with cells and other ECM components [95]. Fibrin is generated by the crosslinking of fibrinogen and thrombin. Fibrin hydrogels’ mechanical characteristics are primarily influenced by fibrinogen concentration, with thrombin playing a less significant role. To investigate antiangiogenic substances, glioblastoma spheroids and endothelial cells were cocultured in a fibrin matrix [95]. Vascular endothelial growth factor (VEGF)-loaded fibrin hydrogels promote neural stem cell proliferation and migration in comparison to fibrin matrix without VEGF or VEGF-loaded collagen hydrogel, highlighting the advantages of fibrin matrices to embed growth factors for prolonged culture time. To enhance its mechanical and biochemical properties, fibrin bioinks have been combined with gelatin, alginate, or HA [357,358]. These combinations have produced a variety of tissue models, including glioblastoma models, cardiac tissues, and dentin-pulp complex. Matrigel, an ECM mixture that may be thermally cured, is generated from murine Engelbreth-Holm-Swarm sarcoma and contains growth factors and proteoglycans in addition to roughly 60% laminin, 30% collagen type IV, and 8% nidogen [357]. It is especially appropriate for BBB simulation due to its resemblance to the composition of the vascular ECM [359]. Matrigel is therefore widely utilized for in vitro vascular development and related investigations. Additionally, Matrigel has been used to create glioblastoma organoids, with the cells exhibiting heterogeneity in stemness and proliferation as well as hypoxic gradients [359].

Synthetic biomaterials including PEG, PU, and Poly(N-isopropylacrylamide) (PNIPAAm) have been employed in glioblastoma studies. PNIPAAm and its composite hydrogel materials are thermos-responsive and exhibit good printability on extrusion-based bioprinters [357,359]. To obtain a nanoscale resolution and post printing dynamic modulations, PNIPAAm embedded with gold nanorods can be printed using multiphoton lithography [360,361]. The primary GSCs cultivated in a PNIPAAm-PEG matrix maintain their stemness for an extended period of time and are simple to remove and re-encapsulate by changing the hydrogel’s temperature. The hydrogel can multiply GSCs into the huge numbers required for screening. Due to its excellent biocompatibility, inert biochemical characteristics, and customizable mechanical qualities, PEG is a well-liked biomaterial for 3D tissue modelling [361].

Self-Assembled Peptides (SAP)-based hydrogels are crosslinked by physical or chemical peptide bonding, resulting in structured nanofibrous-sheets that resemble biological ECM structures [362]. Chains of amino acids known as peptides have inherent biological characteristics. Fibrous SAP hydrogels are potential bioinks for extrusion-based bioprinting because of their adjustable stimuli-responsive gelation processes such as enzymatic triggering and programmable mechanical properties. SAP’s injectability and ability to adapt to irregular forms make it a promising choice for CNS regenerations such as BBB repair or brain tissue repair following glioblastoma resection [362,363].

## 5. Role of Receptors

Receptors play an important role in signal transduction, immunotherapy, and immune responses. GABA consists of three primary receptors, namely GABA-A, GABA-B, and GABA-C, which play a significant role in its physiology [364]. Together, GABA-A and GABA-C receptors are called ligand-gated chloride (Cl_2_^+^) channels, while GABA-B receptors are G-protein coupled metabotropic receptors. GABA-A receptors act as postsynaptic ionotropic receptors [364]. They release GABA and open Cl^−^ channels in the postsynaptic cell, which leads to hyperpolarization. These receptors may either work as synaptic or extra synaptic [365]. In contrast, GABA-B receptors are metabotropic, while G-protein coupled receptors act through a second messenger cascade. GABA-B receptors act either as postsynaptic or presynaptic, leading to the opening of K^+^ channels following GABA release in the presynaptic terminal limits [364]. K^+^ leads to higher gradient hyperpolarization than Cl^−^ when acting in post-synoptical form, staying longer in action compared to GABA-A receptors [362,364].

In comparison with the all three GABA receptors, GABA-A plays a vital role. Hence, the GABA-A receptor is made up of 16 subunits and the receptors facilitate the majority of the inhibition in the vertebrate brain [364]. Five separate subunits were assembled pentamerically, forming a ligand-gated Cl^2+^ ion channel. Due to their gene differentiation, GABA-A receptor subunits are classified as α1–6, β1–3, γ1–3, ρ1–3, d, p, and e. In comparison, the mammalian brain contains the most common combination of GABA-A receptors with two α, two β, and one γ subunits [362]. Homomeric or heteromeric channels are found in GABA-C receptors, which are composed of r1–3 subunits. This feature makes GABA-A receptor in pharmacology and its function to be supreme [365]. Each of the subunits could form different subtype combinations, which lead to other physiologic characteristics and pharmacologic profiles. These combinations of subtypes rely on co-localization on the neuronal membrane [362,364].

The subunits such as α1, γ2, α4, and δ1of GABA-A have received the most attention in TBI research. Their primary function includes the ability to modulate neuronal signals via phasic inhibition and tonic inhibition [365]. The subunit α1 and γ2 GABA-A receptors are involved in phasic inhibition, while tonic inhibition uses α4 and δ1 subunits. Phasic inhibition the decreases postsynaptic cell hyper-excitability, and it also plays an indispensable role in the formation and modulation of gamma (γ) and theta (θ) oscillations. Simultaneously, tonic inhibition provides functions with constant maintenance and time of postsynaptic depolarization. Both these inhibitions are mainly dependent on the amount and speed of GABA release and postsynaptic receptors’ differential action [362,363].

Furthermore, when GABA is released at a higher gradient from presynaptic vesicles, it diffuses quickly across the synaptic space [365]. It acts on α1 and γ2 containing GABA-A receptors leading to phasic inhibition. Alternatively, gradient levels are lower in concentration leading to tonic inhibition by releasing GABA, which acts on extrasynaptic receptors containing α4 and δ1 subunits [365]. The expression of subunits belonging to GABA-A works similarly to glutamate when inducing excitatory signals. Increased expression is found in the subunit GABA α1 and γ2 subunit during the first few hours of TBI hours later decreased by 24 h. These expression changes are blocked by an NMDA receptor blocker named MK-801, which prevents Ca^2+^ influx into the postsynaptic cell and glutamate release after TBI. Some scientists believe Ca^2+^ obstruction may prevent post-TBI apoptosis by mediating the α1 subunit [365].

## 6. Conclusions and Prospects

The use of nanoparticles in the medical area aids in the improvement of illness detection and treatment by raising the sensitivity of diagnostic tools and altering pharmacological characteristics, which increases the effectiveness of the treatments. The capacity of NPs to penetrate the BBB makes these substances a viable treatment option for brain illnesses and diseases, which have been difficult to diagnose and treat for a long time. However, more research is required to ascertain their cytotoxicity and biosorption in clinical situations in order to guarantee the efficacy and usefulness of these particles. Some of these NPs lead to a peroxidation and oxidative stress which decrease in brain function. it is therefore important to prioritize the use of NPs with therapeutic use and low toxicity. Additionally, it is critical to improve the formulation using targeted antibodies to increase their sensitivity to target certain biomarkers. After more research, nanotechnology’s potential for treating brain problems and illnesses will be unbounded. Hence, to assist the design of versatile, biodegradable, and fully integrated implant systems, bio printed organs, nano sensors, and wearables future research efforts should concentrate on device integration and optimization. Future paths in development for the creation of stretchable, biodegradable, and recyclable electronic systems and biomaterials that are anticipated to have a substantial impact on the advancement of brain illness diagnostics and therapy. The development of computational aids like AI and ML accelerates research and development by making it simpler to occur.

## Data Availability

Data will be made available upon request.

## Abbreviations

11-HT	11-Hydrooxytestosterone
ABD	Autoimmune brain diseases
AD	Alzheimer’s disease
ADHD	Attention-deficit/hyperactivity disorder
AI	Artificial Intelligence
ANN	Artificial neural networks
Anti-DNER	Anti-Delta/Noch like endothelial growth factor
Anti-NMDA	Anti-N-methyl D-Aspartate
APDs	Annihilation photodiode detectors
BBB	Blood brain barrier
BCSFB	Blood-cerebrospinal fluid barrier
BD	Brain decode
CNS	Central Nervous System
CSF	blood-cerebrospinal fluid
CT	Computed tomography
DAMPS	Damage associated molecular patterns
DSA	Digital subtraction angiography
EBT	Electron beam tomography
ECM	Extra cellular matrix
EEG	Electro encephalography
EGF	Endothelial growth factor
FDA	Food and drug administration
fNIRS	Functional near infrared spectroscopy
GAD67	Glutamic acid decarboxylase—67
GBM	Glioblastoma
GBM	Glioblastoma multiforme
GlyR	Glycine receptor
HDA	Histamine deacetylase
IC	Intracranial circulatory
IoT	Internet of things
LC	Liquid chromatography
LC-MS/MS	Liquid chromatography tandem mass spectroscopy
MEMS	Microelectrical mechanical systems
ML	Machine Learning
MRI	Magnetic resonance imaging
MS	Multiple sclerosis
NCLS	Neuronal ceroid lipofuscinoses
NDD	Neurodevelopmental disorder
NPs	Nanoparticles
O/W	Oil in water
PAMAM	Polyamidoamine
PD	Parkinson’s disease
PDMS/CNT	Poly-di-methyl siloxane/carbon nanotubes
PEG	Polyethylene glycol
PET	Positron emission tomography
PLGA	Polylactide Glycolic Acid
PNIPAAm	Poly(N-isopropylacrylamide)
PSMA	Prostate specific membrane antigen
PTSD	Post-traumatic stress disorder
PU	Polyurethane
PVN	Perivascular niche
RMT	Receptor-mediated transcytosis
ROS	Reactive oxygen species
SAP	Self-assembled peptides
SiPM	Silicon photomultiplier
TBI	Traumatic brain injury
TOF	Time of flight
TUH	Temple University Hospital
UCH-L1	Ubiquitin C-terminal hydrolase-L1
VEGF	Vascular endothelial growth factor
W/O	Water in oil

## References

[1] Khellaf A., Khan D.Z., Helmy A. (2019). Recent Advances in Traumatic Brain Injury. J. Neurol..

[2] Sonkar R., Jha A., Viswanadh M.K., Burande A.S., Pawde D.M., Patel K.K., Singh M., Koch B., Muthu M.S. (2021). Gold Liposomes for Brain-Targeted Drug Delivery: Formulation and Brain Distribution Kinetics. Mater. Sci. Eng. C.

[3] Hua H., Zhang X., Mu H., Meng Q., Jiang Y., Wang Y., Lu X., Wang A., Liu S., Zhang Y. (2018). RVG29-Modified Docetaxel-Loaded Nanoparticles for Brain-Targeted Glioma Therapy. Int. J. Pharm..

[4] Lin S., Liu J., Li W., Wang D., Huang Y., Jia C., Li Z., Murtaza M., Wang H., Song J. (2019). A Flexible, Robust, and Gel-Free Electroencephalogram Electrode for Noninvasive Brain-Computer Interfaces. Nano Lett..

[5] Peng Y., Zhao Y., Chen Y., Yang Z., Zhang L., Xiao W., Yang J., Guo L., Wu Y. (2018). Dual-Targeting for Brain-Specific Liposomes Drug Delivery System: Synthesis and Preliminary Evaluation. Bioorganic Med. Chem..

[6] Bachiller S., Jiménez-Ferrer I., Paulus A., Yang Y., Swanberg M., Deierborg T., Boza-Serrano A. (2018). Microglia in Neurological Diseases: A Road Map to Brain-Disease Dependent-Inflammatory Response. Front. Cell Neurosci..

[7] Bolte A.C., Dutta A.B., Hurt M.E., Smirnov I., Kovacs M.A., McKee C.A., Ennerfelt H.E., Shapiro D., Nguyen B.H., Frost E.L. (2020). Meningeal Lymphatic Dysfunction Exacerbates Traumatic Brain Injury Pathogenesis. Nat. Commun..

[8] Bhowmick S., D’Mello V., Caruso D., Wallerstein A., Abdul-Muneer P.M. (2019). Impairment of Pericyte-Endothelium Crosstalk Leads to Blood-Brain Barrier Dysfunction Following Traumatic Brain Injury. Exp. Neurol..

[9] Sollmann N., Beer A.J., Kirchhoff F. (2022). SARS-CoV-2 Infection and the Brain: Direct Evidence for Brain Changes in Milder Cases. Signal Transduct. Target. Ther..

[10] Natoli S., Oliveira V., Calabresi P., Maia L.F., Pisani A. (2020). Does SARS-Cov-2 Invade the Brain? Translational Lessons from Animal Models. Eur. J. Neurol..

[11] Barber C.N., Raben D.M. (2019). Lipid Metabolism Crosstalk in the Brain: Glia and Neurons. Front. Cell Neurosci..

[12] Hu W., Jiang J., Xie D., Wang S., Bi K., Duan H., Yang J., He J. (2018). Transient Security Transistors Self-Supported on Biodegradable Natural-Polymer Membranes for Brain-Inspired Neuromorphic Applications. Nanoscale.

[13] Ladak A.A., Enam S.A., Ibrahim M.T. (2019). A Review of the Molecular Mechanisms of Traumatic Brain Injury. World Neurosurg..

[14] Sullan M.J., Asken B.M., Jaffee M.S., DeKosky S.T., Bauer R.M. (2018). Glymphatic System Disruption as a Mediator of Brain Trauma and Chronic Traumatic Encephalopathy. Neurosci. Biobehav. Rev..

[15] Munley K.M., Wade K.L., Pradhan D.S. (2022). Uncovering the Seasonal Brain: Liquid Chromatography-Tandem Mass Spectrometry (LC-MS/MS) as a Biochemical Approach for Studying Seasonal Social Behaviors. Horm. Behav..

[16] Mahjoub I., Mahjoub M.A., Rekik I., Weiner M., Aisen P., Petersen R., Jack C., Jagust W., Trojanowki J., Toga A. (2018). Brain Multiplexes Reveal Morphological Connectional Biomarkers Fingerprinting Late Brain Dementia States. Sci. Rep..

[17] Xie J., Shen Z., Anraku Y., Kataoka K., Chen X. (2019). Nanomaterial-Based Blood-Brain-Barrier (BBB) Crossing Strategies. Biomaterials.

[18] Bao Q., Hu P., Xu Y., Cheng T., Wei C., Pan L., Shi J. (2018). Simultaneous Blood-Brain Barrier Crossing and Protection for Stroke Treatment Based on Edaravone-Loaded Ceria Nanoparticles. ACS Nano.

[19] Manojkumar K., Kandeeban R., Brindha R., Sangeetha V., Saminathan K. (2022). Non-Precious Metal-Based Integrated Electrodes for Overall Alkaline Water Splitting. J. Indian Chem. Soc..

[20] Ramasubramanian B., Reddy M.v., Zaghib K., Armand M., Ramakrishna S. (2021). Growth Mechanism of Micro/Nano Metal Dendrites and Cumulative Strategies for Countering Its Impacts in Metal Ion Batteries: A Review. Nanomaterials.

[21] Brindha R., Mohanraj R., Manojkumar P., Selvam M., Sakthipandi K. (2020). Hybrid Electrochemical Behaviour of La_1−x_CaxMnO_3_ Nano Perovskites and Recycled Polar Interspersed Graphene for Metal-Air Battery System. J. Electrochem. Soc..

[22] Mohanraj R., Brindha R., Kandeeban R., Mahendhar M., Saminathan K., Ayyappadasan G. (2021). Electrochemical Detection of 5-Hydroxytryptamine Using Sustainable SnO2-Graphite Nanocomposite Modified Electrode. Mater. Lett..

[23] Ramasubramanian B., Sundarrajan S., Chellappan V., Reddy M.V., Ramakrishna S., Zaghib K. (2022). Recent Development in Carbon-LiFePO4 Cathodes for Lithium-Ion Batteries: A Mini Review. Batteries.

[24] Kumar K.K., Brindha R., Nandhini M., Selvam M., Saminathan K., Sakthipandi K. (2019). Water-Suspended Graphene as Electrolyte Additive in Zinc-Air Alkaline Battery System. Ionics.

[25] Brindha R., Ajith S.S.R., Nandhini M., Selvam M., Subannajui K., Khotmungkhun K., Sakthipandi K. (2019). Evaluation of Anticorrosive Behaviour of ZnO Nanotetra-Pods on a AZ91-Grade Mg Alloy. Bull. Mater. Sci..

[26] Ramasubramanian B., Chinglenthoiba C., Huiqing X., Xiping N., Hui H.K., Valiyaveettil S., Ramakrishna S., Chellappan V. (2022). Sustainable Fe-MOF@carbon Nanocomposite Electrode for Supercapacitor. Surf. Interfaces.

[27] Kandeeban R., Brindha R., Manojkumar K., Batoo K.M., Raslan E.H., Hadi M., Imran A., Saminathan K. (2021). Revealing the Synergetic Electrocatalyst Behaviour of Kish Graphite Recovered from Polyethylene Plastics. Mater. Lett..

[28] Wang C., Wu B., Wu Y., Song X., Zhang S., Liu Z. (2020). Camouflaging Nanoparticles with Brain Metastatic Tumor Cell Membranes: A New Strategy to Traverse Blood–Brain Barrier for Imaging and Therapy of Brain Tumors. Adv. Funct. Mater..

[29] del Prado-Audelo M.L., Caballero-Florán I.H., Meza-Toledo J.A., Mendoza-Muñoz N., González-Torres M., Florán B., Cortés H., Leyva-Gómez G. (2019). Formulations of Curcumin Nanoparticles for Brain Diseases. Biomolecules.

[30] Teleanu D.M., Chircov C., Grumezescu A.M., Volceanov A., Teleanu R.I. (2018). Impact of Nanoparticles on Brain Health: An Up to Date Overview. J. Clin. Med..

[31] Mukhtar M., Bilal M., Rahdar A., Barani M., Arshad R., Behl T., Brisc C., Banica F., Bungau S. (2020). Nanomaterials for Diagnosis and Treatment of Brain Cancer: Recent Updates. Chemosensors.

[32] Silva G.A. (2018). A New Frontier: The Convergence of Nanotechnology, Brain Machine Interfaces, and Artificial Intelligence. Front. Neurosci..

[33] Schnabel R.B., Hasenfuß G., Buchmann S., Kahl K.G., Aeschbacher S., Osswald S., Angermann C.E. (2021). Heart and Brain Interactions: Pathophysiology and Management of Cardio-Psycho-Neurological Disorders. Herz.

[34] Sakai K., Yamada K. (2018). Machine Learning Studies on Major Brain Diseases: 5-Year Trends of 2014–2018. Jpn. J. Radiol..

[35] Soomro T.A., Zheng L., Afifi A.J., Ali A., Soomro S., Yin M., Gao J. (2022). Image Segmentation for MR Brain Tumor Detection Using Machine Learning: A Review. IEEE Rev. Biomed. Eng..

[36] Burnstock G. (2008). Purinergic Signalling and Disorders of the Central Nervous System. Nat. Rev. Drug Discov..

[37] Deleidi M., Jäggle M., Rubino G. (2015). Immune Ageing, Dysmetabolism and Inflammation in Neurological Diseases. Front. Neurosci..

[38] Wilson M., Andronesi O., Barker P.B., Bartha R., Bizzi A., Bolan P.J., Brindle K.M., Choi I.Y., Cudalbu C., Dydak U. (2019). Methodological Consensus on Clinical Proton MRS of the Brain: Review and Recommendations. Magn. Reson. Med..

[39] Thomi G., Surbek D., Haesler V., Joerger-Messerli M., Schoeberlein A. (2019). Exosomes Derived from Umbilical Cord Mesenchymal Stem Cells Reduce Microglia-Mediated Neuroinflammation in Perinatal Brain Injury. Stem. Cell Res. Ther..

[40] Ceprian M., Fulton D. (2019). Glial Cell AMPA Receptors in Nervous System Health, Injury and Disease. Int. J. Mol. Sci..

[41] Reed C.B., Feltri M.L., Wilson E.R. (2022). Peripheral Glia Diversity. J. Anat..

[42] Donnelly C.R., Andriessen A.S., Chen G., Wang K., Jiang C., Maixner W., Ji R.R. (2020). Central Nervous System Targets: Glial Cell Mechanisms in Chronic Pain. Neurotherapeutics.

[43] Heiss C.N., Olofsson L.E. (2019). The Role of the Gut Microbiota in Development, Function and Disorders of the Central Nervous System and the Enteric Nervous System. J. Neuroendocrinol..

[44] Xu S., Lu J., Shao A., Zhang J.H., Zhang J. (2020). Glial Cells: Role of the Immune Response in Ischemic Stroke. Front. Immunol..

[45] Pöyhönen S., Er S., Domanskyi A., Airavaara M. (2019). Effects of Neurotrophic Factors in Glial Cells in the Central Nervous System: Expression and Properties in Neurodegeneration and Injury. Front. Physiol..

[46] Lillicrap T.P., Santoro A., Marris L., Akerman C.J., Hinton G. (2020). Backpropagation and the Brain. Nat. Rev. Neurosci..

[47] Keller D., Erö C., Markram H. (2018). Cell Densities in the Mouse Brain: A Systematic Review. Front. Neuroanat..

[48] Yang Q.Q., Zhou J.W. (2019). Neuroinflammation in the Central Nervous System: Symphony of Glial Cells. Glia.

[49] Sanson A., Riva M.A. (2020). Anti-Stress Properties of Atypical Antipsychotics. Pharmaceuticals.

[50] Jia Z., Chen X., Tang W., Zhao D., Yu S. (2019). Atypical Functional Connectivity between the Anterior Cingulate Cortex and Other Brain Regions in a Rat Model of Recurrent Headache. Mol. Pain..

[51] Hickman S., Izzy S., Sen P., Morsett L., el Khoury J. (2018). Microglia in Neurodegeneration. Nat. Neurosci..

[52] Hall S., Janelidze S., Surova Y., Widner H., Zetterberg H., Hansson O. (2018). Cerebrospinal Fluid Concentrations of Inflammatory Markers in Parkinson’s Disease and Atypical Parkinsonian Disorders. Sci. Rep..

[53] Sen Z.D., Danyeli L.V., Woelfer M., Lamers F., Wagner G., Sobanski T., Walter M. (2021). Linking Atypical Depression and Insulin Resistance-Related Disorders via Low-Grade Chronic Inflammation: Integrating the Phenotypic, Molecular and Neuroanatomical Dimensions. Brain Behav. Immun..

[54] Cellucci T., van Mater H., Graus F., Muscal E., Gallentine W., Klein-Gitelman M.S., Benseler S.M., Frankovich J., Gorman M.P., van Haren K. (2020). Clinical Approach to the Diagnosis of Autoimmune Encephalitis in the Pediatric Patient. Neurol. Neuroimmunol. Neuroinflamm..

[55] Gialluisi A., Andlauer T.F.M., Mirza-Schreiber N., Moll K., Becker J., Hoffmann P., Ludwig K.U., Czamara D., St Pourcain B., Brandler W. (2019). Genome-Wide Association Scan Identifies New Variants Associated with a Cognitive Predictor of Dyslexia. Transl. Psychiatry.

[56] Li W., Middha M., Bicak M., Sjoberg D.D., Vertosick E., Dahlin A., Häggström C., Hallmans G., Rönn A.C., Stattin P. (2018). Genome-Wide Scan Identifies Role for AOX1 in Prostate Cancer Survival. Eur. Urol..

[57] Barake F., Bravo-Zehnder M., González A. (2022). Progress in the Mechanism of Neuronal Surface P Antigen Modulating Hippocampal Function and Implications for Autoimmune Brain Disease. Curr. Opin. Neurol..

[58] He Z., Xu B., Buxbaum J., Ionita-Laza I. (2019). A Genome-Wide Scan Statistic Framework for Whole-Genome Sequence Data Analysis. Nat. Commun..

[59] van’t Westeinde A., Padilla N., Siqueiros Sanchez M., Fletcher-Sandersjöö S., Kämpe O., Bensing S., Lajic S. (2022). Brain Structure in Autoimmune Addison’s Disease. Cereb. Cortex.

[60] Dai W.L., Bao Y.N., Fan J.F., Ma B., Li S.S., Zhao W.L., Yu B.Y., Liu J.H. (2021). Blockade of Spinal Dopamine D1/D2 Receptor Suppresses Activation of NMDA Receptor through Gαq and Src Kinase to Attenuate Chronic Bone Cancer Pain. J. Adv. Res..

[61] Hamada M., Zaidan B.B., Zaidan A.A. (2018). A Systematic Review for Human EEG Brain Signals Based Emotion Classification, Feature Extraction, Brain Condition, Group Comparison. J. Med. Syst..

[62] Pape K., Tamouza R., Leboyer M., Zipp F. (2019). Immunoneuropsychiatry—Novel Perspectives on Brain Disorders. Nat. Rev. Neurol..

[63] Teleanu D.M., Chircov C., Grumezescu A.M., Volceanov A., Teleanu R.I. (2018). Blood-Brain Delivery Methods Using Nanotechnology. Pharmaceutics.

[64] Florendo M., Figacz A., Srinageshwar B., Sharma A., Swanson D., Dunbar G.L., Rossignol J. (2018). Use of Polyamidoamine Dendrimers in Brain Diseases. Molecules.

[65] Mifflin K., Baker G.B., Kerr B.J. (2018). Effect of Voluntary Wheel Running on Neuroactive Steroid Levels in Murine Experimental Autoimmune Encephalomyelitis. Neurosci. Lett..

[66] Todorov M.I., Paetzold J.C., Schoppe O., Tetteh G., Shit S., Efremov V., Todorov-Völgyi K., Düring M., Dichgans M., Piraud M. (2020). Machine Learning Analysis of Whole Mouse Brain Vasculature. Nat. Methods.

[67] Akamatsu Y., Hanafy K.A. (2020). Cell Death and Recovery in Traumatic Brain Injury. Neurotherapeutics.

[68] Candelario-Jalil E., Dijkhuizen R.M., Magnus T. (2022). Neuroinflammation, Stroke, Blood-Brain Barrier Dysfunction, and Imaging Modalities. Stroke.

[69] Rodriguez-Otormin F., Duro-Castano A., Conejos-Sánchez I., Vicent M.J. (2019). Envisioning the Future of Polymer Therapeutics for Brain Disorders. Wiley Interdiscip. Rev. Nanomed. Nanobiotechnol..

[70] Na S., Wang L.v. (2021). Photoacoustic Computed Tomography for Functional Human Brain Imaging [Invited]. Biomed. Opt. Express.

[71] Hartmann K.G., Schirrmeister R.T., Ball T. (2018). EEG-GAN: Generative Adversarial Networks for Electroencephalograhic (EEG) Brain Signals. arXiv.

[72] Pandit R., Chen L., Götz J. (2019). The Blood-Brain Barrier: Physiology and Strategies for Drug Delivery. Adv. Drug Deliv. Rev..

[73] Xu L., Zhang C., He D., Jiang N., Bai Y., Xin Y. (2020). Rapamycin and MCC950 Modified Gut Microbiota in Experimental Autoimmune Encephalomyelitis Mouse by Brain Gut Axis. Life Sci..

[74] Niu X., Sang H., Wang J. (2021). Naringenin Attenuates Experimental Autoimmune Encephalomyelitis by Protecting the Intact of Blood-Brain Barrier and Controlling Inflammatory Cell Migration. J. Nutr. Biochem..

[75] Shobatake R., Kumazawa A., Koyama N., Takahashi N. (2022). Autoimmune Encephalitis Associated with Anti-N-Methyl-D-Aspartate Receptor and Anti-Hu Antibodies Successfully Treated with Carboplatin and Etoposide for Small-Cell Lung Cancer. Intern. Med..

[76] O’donovan B., Mandel-Brehm C., Vazquez S.E., Liu J., Parent A.V., Anderson M.S., Kassimatis T., Zekeridou A., Hauser S.L., Pittock S.J. (2020). High-Resolution Epitope Mapping of Anti-Hu and Anti-Yo Autoimmunity by Programmable Phage Display. Brain Commun..

[77] Sell J., Haselmann H., Hallermann S., Hust M., Geis C. (2020). Autoimmune Encephalitis: Novel Therapeutic Targets at the Preclinical Level. Expert Opin. Ther. Targets.

[78] Bordonne M., Chawki M.B., Doyen M., Kas A., Guedj E., Tyvaert L., Verger A. (2021). Brain 18F-FDG PET for the Diagnosis of Autoimmune Encephalitis: A Systematic Review and a Meta-Analysis. Eur. J. Nucl. Med. Mol. Imaging.

[79] Shen C.H., Fang G.L., Yang F., Cai M.T., Zheng Y., Fang W., Guo Y., Zhang Y.X., Ding M.P. (2020). Seizures and Risk of Epilepsy in Anti-NMDAR, Anti-LGI1, and Anti-GABABR Encephalitis. Ann. Clin. Transl. Neurol..

[80] Wei Y.C., Tseng J.R., Wu C.L., Su F.C., Weng W.C., Hsu C.C., Chang K.H., Wu C.F., Hsiao I.T., Lin C.P. (2020). Different FDG-PET Metabolic Patterns of Anti-AMPAR and Anti-NMDAR Encephalitis: Case Report and Literature Review. Brain Behav..

[81] Ariño H., Muñoz-Lopetegi A., Martinez-Hernandez E., Armangue T., Rosa-Justicia M., Escudero D., Matos N., Graus F., Sugranyes G., Castro-Fornieles J. (2020). Sleep Disorders in Anti-NMDAR Encephalitis. Neurology.

[82] Seery N., Butzkueven H., O’Brien T.J., Monif M. (2022). Contemporary Advances in Anti-NMDAR Antibody (Ab)-Mediated Encephalitis. Autoimmun. Rev..

[83] Omi T., Kinoshita M., Nishikawa A., Tomioka T., Ohmori K., Fukada K., Matsunaga H. (2018). Clinical Relapse of Anti-AMPA Receptor Encephalitis Associated with Recurrence of Thymoma. Intern. Med..

[84] Wang T., Wen B., Chi Z., Zhao X. (2021). The Well Responsiveness of Drug-Resistant Focal Seizures in Anti-AMPA2 Receptor Encephalitis to Perampanel Treatment. Neurol. Sci..

[85] Mohamadpour M., Whitney K., Bergold P.J. (2019). The Importance of Therapeutic Time Window in the Treatment of Traumatic Brain Injury. Front. Neurosci..

[86] Malik R., Burch D., Bazant M., Ceder G. (2010). Particle Size Dependence of the Ionic Diffusivity. Nano Lett..

[87] Milenkovic J., Hrenovic J., Goic-Barisic I., Tomic M., Djonlagic J., Rajic N. (2014). Synergistic Anti-Biofouling Effect of Ag-Exchanged Zeolite and D-Tyrosine on PVC Composite against the Clinical Isolate of Acinetobacter Baumannii. Biofouling.

[88] Yeung M.S.Y., Djelloul M., Steiner E., Bernard S., Salehpour M., Possnert G., Brundin L., Frisén J. (2019). Dynamics of Oligodendrocyte Generation in Multiple Sclerosis. Nature.

[89] Hauser S.L., Cree B.A.C. (2020). Treatment of Multiple Sclerosis: A Review. Am. J. Med..

[90] McGinley M.P., Goldschmidt C.H., Rae-Grant A.D. (2021). Diagnosis and Treatment of Multiple Sclerosis: A Review. J. Assoc. Med. Am..

[91] Dobson R., Giovannoni G. (2019). Multiple Sclerosis—A Review. Eur. J. Neurol..

[92] Milh M., Roubertoux P., Biba N., Chavany J., Spiga Ghata A., Fulachier C., Collins S.C., Wagner C., Roux J.C., Yalcin B. (2020). A Knock-in Mouse Model for KCNQ2-Related Epileptic Encephalopathy Displays Spontaneous Generalized Seizures and Cognitive Impairment. Epilepsia.

[93] Allers K., Essue B.M., Hackett M.L., Muhunthan J., Anderson C.S., Pickles K., Scheibe F., Jan S. (2015). The Economic Impact of Epilepsy: A Systematic Review. BMC Neurol..

[94] Karoly P.J., Rao V.R., Gregg N.M., Worrell G.A., Bernard C., Cook M.J., Baud M.O. (2021). Cycles in Epilepsy. Nat. Rev. Neurol..

[95] Perkins K.L., Arranz A.M., Yamaguchi Y., Hrabetova S. (2017). Brain Extracellular Space, Hyaluronan, and the Prevention of Epileptic Seizures. Rev. Neurosci..

[96] Helmstaedter C., Witt J.A. (2017). Epilepsy and Cognition—A Bidirectional Relationship?. Seizure.

[97] Kuroda N. (2021). Epilepsy and COVID-19: Updated Evidence and Narrative Review. Epilepsy Behav..

[98] Xu C., Gong Y., Wang Y., Chen Z. (2022). New Advances in Pharmacoresistant Epilepsy towards Precise Management-from Prognosis to Treatments. Pharmacol. Ther..

[99] Bayat A., Bayat M., Rubboli G., Møller R.S. (2021). Epilepsy Syndromes in the First Year of Life and Usefulness of Genetic Testing for Precision Therapy. Genes.

[100] Tsai M.L., Chen C.L., Hsieh K.L.C., Miser J.S., Chang H., Liu Y.L., Wong T.T. (2018). Seizure Characteristics Are Related to Tumor Pathology in Children with Brain Tumors. Epilepsy Res..

[101] Romoli M., Sen A., Parnetti L., Calabresi P., Costa C. (2021). Amyloid-β: A Potential Link between Epilepsy and Cognitive Decline. Nat. Rev. Neurol..

[102] Khambhati A.N., Shafi A., Rao V.R., Chang E.F. (2021). Long-Term Brain Network Reorganization Predicts Responsive Neurostimulation Outcomes for Focal Epilepsy. Sci. Transl. Med..

[103] Larivière S., Royer J., Rodríguez-Cruces R., Paquola C., Caligiuri M.E., Gambardella A., Concha L., Keller S.S., Cendes F., Yasuda C.L. (2022). Structural Network Alterations in Focal and Generalized Epilepsy Assessed in a Worldwide ENIGMA Study Follow Axes of Epilepsy Risk Gene Expression. Nat. Commun..

[104] Vezzani A., Balosso S., Ravizza T. (2019). Neuroinflammatory Pathways as Treatment Targets and Biomarkers in Epilepsy. Nat. Rev. Neurol..

[105] Pitchaimuthu K., Wu Q.Z., Carter O., Nguyen B.N., Ahn S., Egan G.F., McKendrick A.M. (2017). Occipital GABA Levels in Older Adults and Their Relationship to Visual Perceptual Suppression. Sci. Rep..

[106] Mikkelsen M., Singh K.D., Sumner P., Evans C.J. (2016). Comparison of the Repeatability of GABA-Edited Magnetic Resonance Spectroscopy with and without Macromolecule Suppression. Magn. Reson. Med..

[107] Cook E., Hammett S.T., Larsson J. (2016). GABA Predicts Visual Intelligence. Neurosci. Lett..

[108] Derk J., Jones H.E., Como C., Pawlikowski B., Siegenthaler J.A. (2021). Living on the Edge of the CNS: Meninges Cell Diversity in Health and Disease. Front. Cell Neurosci..

[109] Oordt-Speets A.M., Bolijn R., van Hoorn R.C., Bhavsar A., Kyaw M.H. (2018). Global Etiology of Bacterial Meningitis: A Systematic Review and Meta-Analysis. PLoS ONE.

[110] Thanabalasuriar A., Scott B.N.V., Peiseler M., Willson M.E., Zeng Z., Warrener P., Keller A.E., Surewaard B.G.J., Dozier E.A., Korhonen J.T. (2019). Neutrophil Extracellular Traps Confine Pseudomonas Aeruginosa Ocular Biofilms and Restrict Brain Invasion. Cell Host Microbe.

[111] Holdaway I., Reid I., Young N., Thomas M. (2018). Meningitis in Adults: Diagnosis and Management. Intern. Med. J..

[112] Pellegrini L., Albecka A., Mallery D.L., Kellner M.J., Paul D., Carter A.P., James L.C., Lancaster M.A. (2020). SARS-CoV-2 Infects the Brain Choroid Plexus and Disrupts the Blood-CSF Barrier in Human Brain Organoids. Cell Stem Cell.

[113] de Melo G.D., Lazarini F., Levallois S., Hautefort C., Michel V., Larrous F., Verillaud B., Aparicio C., Wagner S., Gheusi G. (2021). COVID-19-Related Anosmia Is Associated with Viral Persistence and Inflammation in Human Olfactory Epithelium and Brain Infection in Hamsters. Sci. Transl. Med..

[114] Wichgers Schreur P.J., van Keulen L., Anjema D., Kant J., Kortekaas J. (2018). Microencephaly in Fetal Piglets Following in Utero Inoculation of Zika Virus Article. Emerg. Microbes Infect..

[115] Subbaiyan R., Ganesan A., Ramasubramanian B. (2022). Self-Potent Anti-Microbial and Anti-Fouling Action of Silver Nanoparticles Derived from Lichen-Associated Bacteria. Appl. Nanosci..

[116] Brindha R., Kandeeban R., Kamal K.S., Manojkumar K., Nithya V., Saminathan K. (2021). Andrographis Paniculata Absorbed ZnO Nanofibers as a Potential Antimicrobial Agent for Biomedical Applications. Adv. Nat. Sci. Nanosci. Nanotechnol..

[117] Ramasubramanian B., Sundarrajan S., Rao R.P., Reddy M.V., Chellappan V., Ramakrishna S. (2022). Novel Low-Carbon Energy Solutions for Powering Emerging Wearables, Smart Textiles, and Medical Devices. Energy Environ. Sci..

[118] Adams Waldorf K.M., Nelson B.R., Stencel-Baerenwald J.E., Studholme C., Kapur R.P., Armistead B., Walker C.L., Merillat S., Vornhagen J., Tisoncik-Go J. (2018). Congenital Zika Virus Infection as a Silent Pathology with Loss of Neurogenic Output in the Fetal Brain. Nat. Med..

[119] Figueiredo C.P., Barros-Aragão F.G.Q., Neris R.L.S., Frost P.S., Soares C., Souza I.N.O., Zeidler J.D., Zamberlan D.C., de Sousa V.L., Souza A.S. (2019). Zika Virus Replicates in Adult Human Brain Tissue and Impairs Synapses and Memory in Mice. Nat. Commun..

[120] Mustafá Y.M., Meuren L.M., Coelho S.V.A., de Arruda L.B. (2019). Pathways Exploited by Flaviviruses to Counteract the Blood-Brain Barrier and Invade the Central Nervous System. Front. Microbiol..

[121] Hasel P., Rose I.V.L., Sadick J.S., Kim R.D., Liddelow S.A. (2021). Neuroinflammatory Astrocyte Subtypes in the Mouse Brain. Nat. Neurosci..

[122] Heneka M.T., McManus R.M., Latz E. (2018). Inflammasome Signalling in Brain Function and Neurodegenerative Disease. Nat. Rev. Neurosci..

[123] Kesika P., Suganthy N., Sivamaruthi B.S., Chaiyasut C. (2021). Role of Gut-Brain Axis, Gut Microbial Composition, and Probiotic Intervention in Alzheimer’s Disease. Life Sci..

[124] Erickson M.A., Banks W.A. (2019). Age-Associated Changes in the Immune System and Blood–Brain Barrier Functions. Int. J. Mol. Sci..

[125] Deloid G.M., Sohal I.S., Lorente L.R., Molina R.M., Pyrgiotakis G., Stevanovic A., Zhang R., McClements D.J., Geitner N.K., Bousfield D.W. (2018). Reducing Intestinal Digestion and Absorption of Fat Using a Nature-Derived Biopolymer: Interference of Triglyceride Hydrolysis by Nanocellulose. ACS Nano.

[126] Komori T. (2020). Updating the Grading Criteria for Adult Diffuse Gliomas: Beyond the WHO2016CNS Classification. Brain Tumor Pathol..

[127] Zhen Y., Reddy V.S., Ramasubramanian B., Ramakrishna S. (2022). Three-Dimensional AgNps@Mxene@PEDOT:PSS Composite Hybrid Foam as a Piezoresistive Pressure Sensor with Ultra-Broad Working Range. J. Mater. Sci..

[128] Bhattacharya A., Choi W.W.Y., Muffat J., Li Y. (2022). Modeling Developmental Brain Diseases Using Human Pluripotent Stem Cells-Derived Brain Organoids – Progress and Perspective. J. Mol. Biol..

[129] Watanabe S., Shirogane Y., Sato Y., Hashiguchi T., Yanagi Y. (2019). New Insights into Measles Virus Brain Infections. Trends Microbiol..

[130] Al-Obaidi M.M.J., Desa M.N.M. (2018). Mechanisms of Blood Brain Barrier Disruption by Different Types of Bacteria, and Bacterial–Host Interactions Facilitate the Bacterial Pathogen Invading the Brain. Cell. Mol. Neurobiol..

[131] Fenster R.J., Lebois L.A.M., Ressler K.J., Suh J. (2018). Brain Circuit Dysfunction in Post-Traumatic Stress Disorder: From Mouse to Man. Nat. Rev. Neurosci..

[132] Betlazar C., Harrison-Brown M., Middleton R.J., Banati R., Liu G.J. (2018). Cellular Sources and Regional Variations in the Expression of the Neuroinflammatory Marker Translocator Protein (TSPO) in the Normal Brain. Int. J. Mol. Sci..

[133] Girgenti M.J., Wang J., Ji D., Cruz D.A., Alvarez V.E., Benedek D., Brady C., Davis D.A., Holtzheimer P.E., Keane T.M. (2020). Transcriptomic Organization of the Human Brain in Post-Traumatic Stress Disorder. Nat. Neurosci..

[134] Bryant R.A. (2019). Post-Traumatic Stress Disorder: A State-of-the-Art Review of Evidence and Challenges. World Psychiatry.

[135] de Erausquin G.A., Snyder H., Carrillo M., Hosseini A.A., Brugha T.S., Seshadri S. (2021). The Chronic Neuropsychiatric Sequelae of COVID-19: The Need for a Prospective Study of Viral Impact on Brain Functioning. Alzheimer’s Dement..

[136] Liu C., Goel P., Kaeser P.S. (2021). Spatial and Temporal Scales of Dopamine Transmission. Nat. Rev. Neurosci..

[137] Fukuyama K., Kato R., Murata M., Shiroyama T., Okada M. (2019). Clozapine Normalizes a Glutamatergic Transmission Abnormality Induced by an Impaired NMDA Receptor in the Thalamocortical Pathway via the Activation of a Group III Metabotropic Glutamate Receptor. Biomolecules.

[138] Etchepare L., Gréa H., Durand P., Bouchet D., Groc L. (2021). NMDA Receptor Membrane Dynamics Tunes the Firing Pattern of Midbrain Dopaminergic Neurons. J. Physiol..

[139] Nesbit M.O., Chai A., Axerio-Cilies P., Phillips A.G., Wang Y.T., Held K. (2022). The Selective Dopamine D1 Receptor Agonist SKF81297 Modulates NMDA Receptor Currents Independently of D1 Receptors. Neuropharmacology.

[140] Pan X., Kaminga A.C., Wen S.W., Wu X., Acheampong K., Liu A. (2019). Dopamine and Dopamine Receptors in Alzheimer’s Disease: A Systematic Review and Network Meta-Analysis. Front. Aging Neurosci..

[141] Umek N., Geršak B., Vintar N., Šoštarič M., Mavri J. (2018). Dopamine Autoxidation Is Controlled by Acidic PH. Front. Mol. Neurosci..

[142] Yang C., Hu K., Wang D., Zubi Y., Lee S.T., Puthongkham P., Mirkin M.V., Venton B.J. (2019). Cavity Carbon-Nanopipette Electrodes for Dopamine Detection. Anal. Chem..

[143] Stępnicki P., Kondej M., Kaczor A.A. (2018). Current Concepts and Treatments of Schizophrenia. Molecules.

[144] Braff D.L. (2015). The Importance of Endophenotypes in Schizophrenia Research. Schizophr. Res..

[145] Cloitre M., Hyland P., Bisson J.I., Brewin C.R., Roberts N.P., Karatzias T., Shevlin M. (2019). ICD-11 Posttraumatic Stress Disorder and Complex Posttraumatic Stress Disorder in the United States: A Population-Based Study. J. Trauma. Stress.

[146] Simon N., Roberts N.P., Lewis C.E., van Gelderen M.J., Bisson J.I. (2019). Associations between Perceived Social Support, Posttraumatic Stress Disorder (PTSD) and Complex PTSD (CPTSD): Implications for Treatment. Eur. J. Psychotraumatology.

[147] Sun L., Sun Z., Wu L., Zhu Z., Zhang F., Shang Z., Jia Y., Gu J., Zhou Y., Wang Y. (2021). Prevalence and Risk Factors for Acute Posttraumatic Stress Disorder during the COVID-19 Outbreak. J. Affect. Disord..

[148] Forte G., Favieri F., Tambelli R., Casagrande M. (2020). COVID-19 Pandemic in the Italian Population: Validation of a Post-Traumatic Stress Disorder Questionnaire and Prevalence of PTSD Symptomatology. Int. J. Environ. Res. Public Health.

[149] Lewis C., Roberts N.P., Gibson S., Bisson J.I. (2020). Dropout from Psychological Therapies for Post-Traumatic Stress Disorder (PTSD) in Adults: Systematic Review and Meta-Analysis. Eur. J. Psychotraumatology.

[150] Labbadia J., Morimoto R.I. (2013). Huntington’s Disease: Underlying Molecular Mechanisms and Emerging Concepts. Trends Biochem. Sci..

[151] Rauf A., Badoni H., Abu-Izneid T., Olatunde A., Rahman M.M., Painuli S., Semwal P., Wilairatana P., Mubarak M.S. (2022). Neuroinflammatory Markers: Key Indicators in the Pathology of Neurodegenerative Diseases. Molecules.

[152] Voet S., Srinivasan S., Lamkanfi M., Loo G. (2019). van Inflammasomes in Neuroinflammatory and Neurodegenerative Diseases. EMBO Mol. Med..

[153] Hou Y., Dan X., Babbar M., Wei Y., Hasselbalch S.G., Croteau D.L., Bohr V.A. (2019). Ageing as a Risk Factor for Neurodegenerative Disease. Nat. Rev. Neurol..

[154] Slanzi A., Iannoto G., Rossi B., Zenaro E., Constantin G. (2020). In Vitro Models of Neurodegenerative Diseases. Front. Cell Dev. Biol..

[155] Vicente Miranda H., El-Agnaf O.M.A., Outeiro T.F. (2016). Glycation in Parkinson’s Disease and Alzheimer’s Disease. Mov. Disord..

[156] Wisniewski T., Goñi F. (2015). Immunotherapeutic Approaches for Alzheimer’s Disease. Neuron.

[157] Bondi M.W., Edmonds E.C., Salmon D.P. (2017). Alzheimer’s Disease: Past, Present, and Future. J. Int. Neuropsychol. Soc..

[158] Barnat M., Capizzi M., Aparicio E., Boluda S., Wennagel D., Kacher R., Kassem R., Lenoir S., Agasse F., Bra B.Y. (2020). Huntington’s Disease Alters Human Neurodevelopment. Science.

[159] Shannon K.M., Fraint A. (2015). Therapeutic Advances in Huntington’s Disease. Mov. Disord..

[160] McColgan P., Tabrizi S.J. (2018). Huntington’s Disease: A Clinical Review. Eur. J. Neurol..

[161] Astle D.E., Holmes J., Kievit R., Gathercole S.E. (2022). Annual Research Review: The Transdiagnostic Revolution in Neurodevelopmental Disorders. J. Child. Psychol. Psychiatry.

[162] Crawley J.N. (2022). Translational Animal Models of Autism and Neurodevelopmental Disorders. Dialogues Clin. Neurosci..

[163] Stoodley C.J. (2015). The Cerebellum and Neurodevelopmental Disorders. Cerebellum.

[164] Summers J., Baribeau D., Mockford M., Goldhopf L., Ambrozewicz P., Szatmari P., Vorstman J. (2020). Supporting Children with Neurodevelopmental Disorders during the COVID-19 Pandemic. J. Am. Acad. Child Adolesc. Psychiatry.

[165] Wang T., Hoekzema K., Vecchio D., Wu H., Sulovari A., Coe B.P., Gillentine M.A., Wilfert A.B., Perez-Jurado L.A., Kvarnung M. (2020). Large-Scale Targeted Sequencing Identifies Risk Genes for Neurodevelopmental Disorders. Nat. Commun..

[166] Han V.X., Patel S., Jones H.F., Nielsen T.C., Mohammad S.S., Hofer M.J., Gold W., Brilot F., Lain S.J., Nassar N. (2021). Maternal Acute and Chronic Inflammation in Pregnancy Is Associated with Common Neurodevelopmental Disorders: A Systematic Review. Transl. Psychiatry.

[167] Zengeler K.E., Lukens J.R. (2021). Innate Immunity at the Crossroads of Healthy Brain Maturation and Neurodevelopmental Disorders. Nat. Rev. Immunol..

[168] Chen G.T., Geschwind D.H. (2022). Challenges and Opportunities for Precision Medicine in Neurodevelopmental Disorders. Adv. Drug Deliv. Rev..

[169] Zeidan J., Fombonne E., Scorah J., Ibrahim A., Durkin M.S., Saxena S., Yusuf A., Shih A., Elsabbagh M. (2022). Global Prevalence of Autism: A Systematic Review Update. Autism Res..

[170] Marotta R., Risoleo M.C., Messina G., Parisi L., Carotenuto M., Vetri L., Roccella M. (2020). The Neurochemistry of Autism. Brain Sci..

[171] Snowling M.J., Hulme C., Nation K. (2020). Defining and Understanding Dyslexia: Past, Present and Future. Oxf. Rev. Educ..

[172] Misiak B., Kowalski K., Piotrowski P., Grąźlewski T., Samochowiec J. (2022). Neurodevelopmental Aspects of Adverse Childhood Experiences in Psychosis: Relevance of the Allostatic Load Concept. Psychoneuroendocrinology.

[173] Iadecola C., Anrather J. (2011). Stroke Research at a Crossroad: Asking the Brain for Directions. Nat. Neurosci..

[174] Thakor J., Ahadian S., Niakan A., Banton E., Nasrollahi F., Hasani-Sadrabadi M.M., Khademhosseini A. (2020). Engineered Hydrogels for Brain Tumor Culture and Therapy. Bio-Des. Manuf..

[175] Yang C., Hawkins K.E., Doré S., Candelario-Jalil E. (2019). Neuroinflammatory Mechanisms of Blood-Brain Barrier Damage in Ischemic Stroke. Am. J. Physiol. Cell Physiol..

[176] Kreienkamp H.J., Wagner M., Weigand H., McConkie-Rossell A., McDonald M., Keren B., Mignot C., Gauthier J., Soucy J.F., Michaud J.L. (2022). Variant-Specific Effects Define the Phenotypic Spectrum of HNRNPH2-Associated Neurodevelopmental Disorders in Males. Hum. Genet..

[177] Tan A.C., Ashley D.M., López G.Y., Malinzak M., Friedman H.S., Khasraw M. (2020). Management of Glioblastoma: State of the Art and Future Directions. CA Cancer J. Clin..

[178] le Rhun E., Preusser M., Roth P., Reardon D.A., van den Bent M., Wen P., Reifenberger G., Weller M. (2019). Molecular Targeted Therapy of Glioblastoma. Cancer Treat. Rev..

[179] Birzu C., French P., Caccese M., Cerretti G., Idbaih A., Zagonel V., Lombardi G. (2020). Recurrent Glioblastoma: From Molecular Landscape to New Treatment Perspectives. Cancers.

[180] Hilf N., Kuttruff-Coqui S., Frenzel K., Bukur V., Stevanović S., Gouttefangeas C., Platten M., Tabatabai G., Dutoit V., van der Burg S.H. (2018). Actively Personalized Vaccination Trial for Newly Diagnosed Glioblastoma. Nature.

[181] Jackson C.M., Choi J., Lim M. (2019). Mechanisms of Immunotherapy Resistance: Lessons from Glioblastoma. Nat. Immunol..

[182] Venkataramani V., Yang Y., Schubert M.C., Reyhan E., Tetzlaff S.K., Wißmann N., Botz M., Soyka S.J., Beretta C.A., Pramatarov R.L. (2022). Glioblastoma Hijacks Neuronal Mechanisms for Brain Invasion. Cell.

[183] Suzuka J., Tsuda M., Wang L., Kohsaka S., Kishida K., Semba S., Sugino H., Aburatani S., Frauenlob M., Kurokawa T. (2021). Rapid Reprogramming of Tumour Cells into Cancer Stem Cells on Double-Network Hydrogels. Nat. Biomed. Eng..

[184] Iranmanesh Y., Jiang B., Favour O.C., Dou Z., Wu J., Li J., Sun C. (2021). Mitochondria’s Role in the Maintenance of Cancer Stem Cells in Glioblastoma. Front. Oncol..

[185] Su C., Zhang J., Yarden Y., Fu L. (2021). The Key Roles of Cancer Stem Cell-Derived Extracellular Vesicles. Signal Transduct. Target. Ther..

[186] Biserova K., Jakovlevs A., Uljanovs R., Strumfa I. (2021). Cancer Stem Cells: Significance in Origin, Pathogenesis and Treatment of Glioblastoma. Cells.

[187] Jiang J.S., Hua Y., Zhou X.J., Shen D.D., Shi J.L., Ge M., Geng Q.N., Jia Z.Z. (2019). Quantitative Assessment of Tumor Cell Proliferation in Brain Gliomas with Dynamic Contrast-Enhanced MRI. Acad. Radiol..

[188] Perez A., Huse J.T. (2021). The Evolving Classification of Diffuse Gliomas: World Health Organization Updates for 2021. Curr. Neurol. Neurosci. Rep..

[189] Salehinejad M.A., Ghanavati E., Glinski B., Hallajian A.H., Azarkolah A. (2022). A Systematic Review of Randomized Controlled Trials on Efficacy and Safety of Transcranial Direct Current Stimulation in Major Neurodevelopmental Disorders: ADHD, Autism, and Dyslexia. Brain Behav..

[190] Hwang E.I., Sayour E.J., Flores C.T., Grant G., Wechsler-Reya R., Hoang-Minh L.B., Kieran M.W., Salcido J., Prins R.M., Figg J.W. (2022). The Current Landscape of Immunotherapy for Pediatric Brain Tumors. Nat. Cancer.

[191] Iroh Tam P.Y., Bendel C.M. (2017). Diagnostics for Neonatal Sepsis: Current Approaches and Future Directions. Pediatric Res..

[192] Weiss Z.F., Leon A., Koo S. (2021). The Evolving Landscape of Fungal Diagnostics, Current and Emerging Microbiological Approaches. J. Fungi.

[193] Altaheri H., Muhammad G., Alsulaiman M., Amin S.U., Altuwaijri G.A., Abdul W., Bencherif M.A., Faisal M. (2021). Deep Learning Techniques for Classification of Electroencephalogram (EEG) Motor Imagery (MI) Signals: A Review. Neural Comput. Appl..

[194] Halim Z., Rehan M. (2020). On Identification of Driving-Induced Stress Using Electroencephalogram Signals: A Framework Based on Wearable Safety-Critical Scheme and Machine Learning. Inf. Fusion.

[195] Craik A., He Y., Contreras-Vidal J.L. (2019). Deep Learning for Electroencephalogram (EEG) Classification Tasks: A Review. J. Neural. Eng..

[196] Supriya S., Siuly S., Wang H., Zhang Y. (2020). Automated Epilepsy Detection Techniques from Electroencephalogram Signals: A Review Study. Health Inf. Sci. Syst..

[197] Gemein L.A.W., Schirrmeister R.T., Chrabąszcz P., Wilson D., Boedecker J., Schulze-Bonhage A., Hutter F., Ball T. (2020). Machine-Learning-Based Diagnostics of EEG Pathology. Neuroimage.

[198] Barshutina M.N., Volkov V.S., Arsenin A.V., Yakubovsky D.I., Melezhik A.V., Blokhin A.N., Tkachev A.G., Lopachev A.V., Kondrashov V.A. (2021). Biocompatible, Electroconductive, and Highly Stretchable Hybrid Silicone Composites Based on Few-Layer Graphene and Cnts. Nanomaterials.

[199] Jiang X., Bian G.B., Tian Z. (2019). Removal of Artifacts from EEG Signals: A Review. Sensors.

[200] Acharya U.R., Hagiwara Y., Deshpande S.N., Suren S., Koh J.E.W., Oh S.L., Arunkumar N., Ciaccio E.J., Lim C.M. (2019). Characterization of Focal EEG Signals: A Review. Future Gener. Comput. Syst..

[201] Nam J., Lim H.K., Kim N.H., Park J.K., Kang E.S., Kim Y.T., Heo C., Lee O.S., Kim S.G., Yun W.S. (2020). Supramolecular Peptide Hydrogel-Based Soft Neural Interface Augments Brain Signals through a Three-Dimensional Electrical Network. ACS Nano.

[202] Wan Z., Yang R., Huang M., Zeng N., Liu X. (2021). A Review on Transfer Learning in EEG Signal Analysis. Neurocomputing.

[203] Namazi H., Menon A., Krejcar O. (2021). Analysis of the Correlation between Static Visual Stimuli, Eye Movements, and Brain Signals. Fluct. Noise Lett..

[204] Khosla A., Khandnor P., Chand T. (2020). A Comparative Analysis of Signal Processing and Classification Methods for Different Applications Based on EEG Signals. Biocybern. Biomed. Eng..

[205] Salem Ghahfarrokhi S., Khodadadi H. (2020). Human Brain Tumor Diagnosis Using the Combination of the Complexity Measures and Texture Features through Magnetic Resonance Image. Biomed. Signal. Process. Control..

[206] Elf K., Ronne-Engström E., Semnic R., Rostami-Berglund E., Sundblom J., Zetterling M. (2019). Continuous EEG Monitoring after Brain Tumor Surgery. Acta Neurochir..

[207] Khan M.A., Ashraf I., Alhaisoni M., Damaševičius R., Scherer R., Rehman A., Bukhari S.A.C. (2020). Multimodal Brain Tumor Classification Using Deep Learning and Robust Feature Selection: A Machine Learning Application for Radiologists. Diagnostics.

[208] Amin J., Sharif M., Haldorai A., Yasmin M., Nayak R.S. (2021). Brain Tumor Detection and Classification Using Machine Learning: A Comprehensive Survey. Complex Intell. Syst..

[209] Merk T., Peterson V., Köhler R., Haufe S., Richardson R.M., Neumann W.J. (2022). Machine Learning Based Brain Signal Decoding for Intelligent Adaptive Deep Brain Stimulation. Exp. Neurol..

[210] Chen Y.H., op de Beeck M., Vanderheyden L., Carrette E., Mihajlović V., Vanstreels K., Grundlehner B., Gadeyne S., Boon P., van Hoof C. (2014). Soft, Comfortable Polymer Dry Electrodes for High Quality ECG and EEG Recording. Sensors.

[211] Wang L., Dou W., Chen J., Lu K., Zhang F., Abdulaziz M., Su W., Li A., Xu C., Sun Y. (2020). A CNT-PDMS Wearable Device for Simultaneous Measurement of Wrist Pulse Pressure and Cardiac Electrical Activity. Mater. Sci. Eng. C.

[212] Kirbas Cilingir E., Seven E.S., Zhou Y., Walters B.M., Mintz K.J., Pandey R.R., Wikramanayake A.H., Chusuei C.C., Vanni S., Graham R.M. (2021). Metformin Derived Carbon Dots: Highly Biocompatible Fluorescent Nanomaterials as Mitochondrial Targeting and Blood-Brain Barrier Penetrating Biomarkers. J. Colloid Interface Sci..

[213] Furtado D., Björnmalm M., Ayton S., Bush A.I., Kempe K., Caruso F. (2018). Overcoming the Blood–Brain Barrier: The Role of Nanomaterials in Treating Neurological Diseases. Adv. Mater..

[214] Zottel A., Paska A.V., Jovčevska I. (2019). Nanotechnology Meets Oncology: Nanomaterials in Brain Cancer Research, Diagnosis and Therapy. Materials.

[215] He K., Liu Y., Wang M., Chen G., Jiang Y., Yu J., Wan C., Qi D., Xiao M., Leow W.R. (2020). An Artificial Somatic Reflex Arc. Adv. Mater..

[216] Lee S., Jeong B., Kim M., Jang R., Paik W., Kang J., Chung W.J., Hong G.S., Kim N. (2022). Emergency Triage of Brain Computed Tomography via Anomaly Detection with a Deep Generative Model. Nat. Commun..

[217] Fletcher’sandersjöö A., Tatter C., Yang L., Pontén E., Boman M., Lassarén P., Forsberg S., Grönlund I., Tidehag V., Rubenson-Wahlin R. (2022). Stockholm Score of Lesion Detection on Computed Tomography Following Mild Traumatic Brain Injury (SELECT-TBI): Study Protocol for a Multicentre, Retrospective, Observational Cohort Study. BMJ Open.

[218] Yokoyama Y., Yamada Y., Kosugi K., Yamada M., Narita K., Nakahara T., Fujiwara H., Toda M., Jinzaki M. (2021). Effect of Gravity on Brain Structure as Indicated on Upright Computed Tomography. Sci. Rep..

[219] Vidhya V., Gudigar A., Raghavendra U., Hegde A., Menon G.R., Molinari F., Ciaccio E.J., Acharya U.R. (2021). Automated Detection and Screening of Traumatic Brain Injury (TBI) Using Computed Tomography Images: A Comprehensive Review and Future Perspectives. Int. J. Environ. Res. Public Health.

[220] Aqeel S., Gupta A., Singh L. (2022). A Review on Unknown Repercussions Associated with Metallic Nanoparticles and Their Rectification Techniques. Curr. Nanomater..

[221] Hamidian K., Sarani M., Sheikhi E., Khatami M. (2022). Cytotoxicity Evaluation of Green Synthesized ZnO and Ag-Doped ZnO Nanoparticles on Brain Glioblastoma Cells. J. Mol. Struct..

[222] Gravesteijn B.Y., Nieboer D., Ercole A., Lingsma H.F., Nelson D., van Calster B., Steyerberg E.W., Åkerlund C., Amrein K., Andelic N. (2020). Machine Learning Algorithms Performed No Better than Regression Models for Prognostication in Traumatic Brain Injury. J. Clin. Epidemiol..

[223] Huang Y., Sun X., Jiang H., Yu S., Robins C., Armstrong M.J., Li R., Mei Z., Shi X., Gerasimov E.S. (2021). A Machine Learning Approach to Brain Epigenetic Analysis Reveals Kinases Associated with Alzheimer’s Disease. Nat. Commun..

[224] Kim D., Jeong Y.Y., Jon S. (2010). A Drug-Loaded Aptamer—Gold Nanoparticle Bioconjugate for Combined Ct Imaging and Therapy of Prostate Cancer. ACS Nano.

[225] Falahati M., Attar F., Sharifi M., Saboury A.A., Salihi A., Aziz F.M., Kostova I., Burda C., Priecel P., Lopez-Sanchez J.A. (2020). Gold Nanomaterials as Key Suppliers in Biological and Chemical Sensing, Catalysis, and Medicine. Biochim. Biophys. Acta Gen. Subj..

[226] Li C.H., Kuo T.R., Su H.J., Lai W.Y., Yang P.C., Chen J.S., Wang D.Y., Wu Y.C., Chen C.C. (2015). Fluorescence-Guided Probes of Aptamer-Targeted Gold Nanoparticles with Computed Tomography Imaging Accesses for in Vivo Tumor Resection. Sci. Rep..

[227] Snelling B.M., Sur S., Shah S.S., Khandelwal P., Caplan J., Haniff R., Starke R.M., Yavagal D.R., Peterson E.C. (2018). Transradial Cerebral Angiography: Techniques and Outcomes. J. Neurointerv. Surg..

[228] Kumar G., Dumitrascu O.M., Chiang C.C., O’Carroll C.B., Alexandrov A.v. (2018). Prediction of Delayed Cerebral Ischemia with Cerebral Angiography: A Meta-Analysis. Neurocritical Care.

[229] Chen S.H., Sur S., Sedighim S., Kassi A., Yavagal D., Peterson E.C., Starke R.M. (2019). Utility of Diagnostic Cerebral Angiography in the Management of Suspected Central Nervous System Vasculitis. J. Clin. Neurosci..

[230] Sajja K.C., Sweid A., al Saiegh F., Chalouhi N., Avery M.B., Schmidt R.F., Tjoumakaris S.I., Gooch M.R., Herial N., Abbas R. (2020). Endovascular Robotic: Feasibility and Proof of Principle for Diagnostic Cerebral Angiography and Carotid Artery Stenting. J. Neurointerv. Surg..

[231] Darsaut T.E., Derksen C., Farzin B., Keough M.B., Fahed R., Boisseau W., Letourneau-Guillon L., Januel A.C., Weill A., Roy D. (2021). Reliability of the Diagnosis of Cerebral Vasospasm Using Catheter Cerebral Angiography: A Systematic Review and Inter- and Intraobserver Study. Am. J. Neuroradiol..

[232] Zhang M., Zhang C., Wu X., Cao X., Young G.S., Chen H., Xu X. (2020). A Neural Network Approach to Segment Brain Blood Vessels in Digital Subtraction Angiography. Comput. Methods Programs Biomed..

[233] Schöll M., Lockhart S.N., Schonhaut D.R., O’Neil J.P., Janabi M., Ossenkoppele R., Baker S.L., Vogel J.W., Faria J., Schwimmer H.D. (2016). PET Imaging of Tau Deposition in the Aging Human Brain. Neuron.

[234] Islam J., Zhang Y. (2020). GAN-Based Synthetic Brain PET Image Generation. Brain Inform..

[235] Sehlin D., Syvänen S., Ballanger B., Barthel H., Bischof G.N., Boche D., Boecker H., Bohn K.P., Borghammer P., Cross D. (2019). Engineered Antibodies: New Possibilities for Brain PET?. Eur. J. Nucl. Med. Mol. Imaging.

[236] Ouerghi H., Mourali O., Zagrouba E. (2018). Non-Subsampled Shearlet Transform Based MRI and PET Brain Image Fusion Using Simplified Pulse Coupled Neural Network and Weight Local Features in YIQ Colour Space. IET Image Process.

[237] Houghton S., Kyron M., Lawrence D., Hunter S.C., Hattie J., Carroll A., Zadow C., Chen W. (2022). Longitudinal Trajectories of Mental Health and Loneliness for Australian Adolescents With-or-without Neurodevelopmental Disorders: The Impact of COVID-19 School Lockdowns. J. Child Psychol. Psychiatry.

[238] Mecheter I., Alic L., Abbod M., Amira A., Ji J. (2020). MR Image-Based Attenuation Correction of Brain PET Imaging: Review of Literature on Machine Learning Approaches for Segmentation. J. Digit. Imaging.

[239] Ladefoged C.N., Marner L., Hindsholm A., Law I., Højgaard L., Andersen F.L. (2019). Deep Learning Based Attenuation Correction of PET/MRI in Pediatric Brain Tumor Patients: Evaluation in a Clinical Setting. Front. Neurosci..

[240] Laurencin C., Lancelot S., Gobert F., Redouté J., Mérida I., Iecker T., Liger F., Irace Z., Greusard E., Lamberet L. (2021). Modeling [^11^C]Yohimbine PET Human Brain Kinetics with Test-Retest Reliability, Competition Sensitivity Studies and Search for a Suitable Reference Region. Neuroimage.

[241] Beck K., Arumuham A., Brugger S., McCutcheon R.A., Veronese M., Santangelo B., McGinnity C.J., Dunn J., Kaar S., Singh N. (2022). The Association between N-Methyl-d-Aspartate Receptor Availability and Glutamate Levels: A Multi-Modal PET-MR Brain Imaging Study in First-Episode Psychosis and Healthy Controls. J. Psychopharmacol..

[242] Won J.Y., Park H., Lee S., Son J.W., Chung Y., Ko G.B., Kim K.Y., Song J., Seo S., Ryu Y. (2021). Development and Initial Results of a Brain PET Insert for Simultaneous 7-Tesla PET/MRI Using an FPGA-Only Signal Digitization Method. IEEE Trans. Med. Imaging.

[243] Slomka P.J., Pan T., Germano G. (2016). Recent Advances and Future Progress in PET Instrumentation. Semin. Nucl. Med..

[244] Huang Z.L., Zhang Z., Qu W.M. (2014). Roles of Adenosine and Its Receptors in Sleep–Wake Regulation. Int. Rev. Neurobiol..

[245] Bahadure N.B., Ray A.K., Thethi H.P. (2018). Comparative Approach of MRI-Based Brain Tumor Segmentation and Classification Using Genetic Algorithm. J. Digit. Imaging.

[246] Mzoughi H., Njeh I., Wali A., Slima M.B., BenHamida A., Mhiri C., Mahfoudhe K.B. (2020). Deep Multi-Scale 3D Convolutional Neural Network (CNN) for MRI Gliomas Brain Tumor Classification. J. Digit. Imaging.

[247] Almansory K.O., Fraioli F. (2019). Combined PET/MRI in Brain Glioma Imaging. Br. J. Hosp. Med..

[248] Badža M.M., Barjaktarović M.C. (2020). Classification of Brain Tumors from MRI Images Using a Convolutional Neural Network. Appl. Sci..

[249] Chen J., Zhang R., Tao C., Huang X., Chen Z., Li X., Zhou J., Zeng Q., Zhao B., Yuan M. (2020). CuS–NiS2 Nanomaterials for MRI Guided Phototherapy of Gastric Carcinoma via Triggering Mitochondria-Mediated Apoptosis and MLKL/CAPG-Mediated Necroptosis. Nanotoxicology.

[250] Li H., Yang S., Hui D., Hong R. (2020). Progress in Magnetic Fe_3_O_4_ Nanomaterials in Magnetic Resonance Imaging. Nanotechnol. Rev..

[251] Arunkumar N., Mohammed M.A., Mostafa S.A., Ibrahim D.A., Rodrigues J.J.P.C., de Albuquerque V.H.C. (2020). Fully Automatic Model-Based Segmentation and Classification Approach for MRI Brain Tumor Using Artificial Neural Networks. Concurr. Comput..

[252] Abd-Ellah M.K., Awad A.I., Khalaf A.A.M., Hamed H.F.A. (2019). A Review on Brain Tumor Diagnosis from MRI Images: Practical Implications, Key Achievements, and Lessons Learned. Magn. Reson. Imaging.

[253] Wadhwa A., Bhardwaj A., Singh Verma V. (2019). A Review on Brain Tumor Segmentation of MRI Images. Magn. Reson. Imaging.

[254] Wood E.A., Stopka S.A., Zhang L., Mattson S., Maasz G., Pirger Z., Vertes A. (2021). Neuropeptide Localization in Lymnaea Stagnalis: From the Central Nervous System to Subcellular Compartments. Front. Mol. Neurosci..

[255] Syslová K., Rambousek L., Kuzma M., Najmanová V., Bubeníková-Valešová V., Šlamberová R., Kačer P. (2011). Monitoring of Dopamine and Its Metabolites in Brain Microdialysates: Method Combining Freeze-Drying with Liquid Chromatography–Tandem Mass Spectrometry. J. Chromatogr. A.

[256] Yücel M.A., Selb J.J., Huppert T.J., Franceschini M.A., Boas D.A. (2017). Functional Near Infrared Spectroscopy: Enabling Routine Functional Brain Imaging. Curr. Opin. Biomed. Eng..

[257] Ayaz H., Onaral B., Izzetoglu K., Shewokis P.A., Mckendrick R., Parasuraman R. (2013). Continuous Monitoring of Brain Dynamics with Functional near Infrared Spectroscopy as a Tool for Neuroergonomic Research: Empirical Examples and a Technological Development. Front. Hum. Neurosci..

[258] Lloyd-Fox S., Blasi A., Elwell C.E. (2010). Illuminating the Developing Brain: The Past, Present and Future of Functional near Infrared Spectroscopy. Neurosci. Biobehav. Rev..

[259] Sajedi H., Pardakhti N. (2019). Age Prediction Based on Brain MRI Image: A Survey. J. Med. Syst..

[260] Cutini S., Brigadoi S. (2014). Unleashing the Future Potential of Functional Near-Infrared Spectroscopy in Brain Sciences. J. Neurosci. Methods.

[261] Kirtane A.R., Verma M., Karandikar P., Furin J., Langer R., Traverso G. (2021). Nanotechnology Approaches for Global Infectious Diseases. Nat. Nanotechnol..

[262] Kwon S., Yoo K.H., Sym S.J., Khang D. (2019). Mesenchymal Stem Cell Therapy Assisted by Nanotechnology: A Possible Combinational Treatment for Brain Tumor and Central Nerve Regeneration. Int. J. Nanomed..

[263] Zheng M., Tao W., Zou Y., Farokhzad O.C., Shi B. (2018). Nanotechnology-Based Strategies for SiRNA Brain Delivery for Disease Therapy. Trends Biotechnol..

[264] Agrawal M., Saraf S., Saraf S., Antimisiaris S.G., Hamano N., Li S.D., Chougule M., Shoyele S.A., Gupta U., Ajazuddin (2018). Recent Advancements in the Field of Nanotechnology for the Delivery of Anti-Alzheimer Drug in the Brain Region. Expert Opin. Drug Deliv..

[265] Tan M.S.A., Parekh H.S., Pandey P., Siskind D.J., Falconer J.R. (2020). Nose-to-Brain Delivery of Antipsychotics Using Nanotechnology: A Review. Expert Opin. Drug Deliv..

[266] Moura R.P., Martins C., Pinto S., Sousa F., Sarmento B. (2019). Blood-Brain Barrier Receptors and Transporters: An Insight on Their Function and How to Exploit Them through Nanotechnology. Expert Opin. Drug Deliv..

[267] Kanazawa T., Kurano T., Ibaraki H., Takashima Y., Suzuki T., Seta Y. (2019). Therapeutic Effects in a Transient Middle Cerebral Artery Occlusion Rat Model by Nose-To-Brain Delivery of Anti-TNF-Alpha SiRNA with Cell-Penetrating Peptide-Modified Polymer Micelles. Pharmaceutics.

[268] Pokharkar V., Suryawanshi S., Dhapte-Pawar V. (2019). Exploring Micellar-Based Polymeric Systems for Effective Nose-to-Brain Drug Delivery as Potential Neurotherapeutics. Drug Deliv. Transl. Res..

[269] Gauro R., Nandave M., Jain V.K., Jain K. (2021). Advances in Dendrimer-Mediated Targeted Drug Delivery to the Brain. J. Nanoparticle Res..

[270] Gothwal A., Kumar H., Nakhate K.T., Ajazuddin, Dutta A., Borah A., Gupta U. (2019). Lactoferrin Coupled Lower Generation PAMAM Dendrimers for Brain Targeted Delivery of Memantine in Aluminum-Chloride-Induced Alzheimer’s Disease in Mice. Bioconjug. Chem..

[271] Bonasia C.G., Abdulahad W.H., Rutgers A., Heeringa P., Bos N.A. (2021). B Cell Activation and Escape of Tolerance Checkpoints: Recent Insights from Studying Autoreactive B Cells. Cells.

[272] Zucca F.A., Vanna R., Cupaioli F.A., Bellei C., de Palma A., di Silvestre D., Mauri P., Grassi S., Prinetti A., Casella L. (2018). Neuromelanin Organelles Are Specialized Autolysosomes That Accumulate Undegraded Proteins and Lipids in Aging Human Brain and Are Likely Involved in Parkinson’s Disease. NPJ Parkinson’s Dis..

[273] You Y., Ikezu T. (2019). Emerging Roles of Extracellular Vesicles in Neurodegenerative Disorders. Neurobiol. Dis..

[274] Wang Y., Fathali H., Mishra D., Olsson T., Keighron J.D., Skibicka K.P., Cans A.S. (2019). Counting the Number of Glutamate Molecules in Single Synaptic Vesicles. J. Am. Chem. Soc..

[275] Ramos-Zaldívar H.M., Polakovicova I., Salas-Huenuleo E., Corvalán A.H., Kogan M.J., Yefi C.P., Andia M.E. (2022). Extracellular Vesicles through the Blood–Brain Barrier: A Review. Fluids Barriers CNS.

[276] Pashirova T.N., Zueva I.V., Petrov K.A., Lukashenko S.S., Nizameev I.R., Kulik N.V., Voloshina A.D., Almasy L., Kadirov M.K., Masson P. (2018). Mixed Cationic Liposomes for Brain Delivery of Drugs by the Intranasal Route: The Acetylcholinesterase Reactivator 2-PAM as Encapsulated Drug Model. Colloids Surf. B Biointerfaces.

[277] Hong S.S., Oh K.T., Choi H.G., Lim S.J. (2019). Liposomal Formulations for Nose-to-Brain Delivery: Recent Advances and Future Perspectives. Pharmaceutics.

[278] Saint-Pol J., Gosselet F., Duban-Deweer S., Pottiez G., Karamanos Y. (2020). Targeting and Crossing the Blood-Brain Barrier with Extracellular Vesicles. Cells.

[279] Gharbavi M., Amani J., Kheiri-Manjili H., Danafar H., Sharafi A. (2018). Niosome: A Promising Nanocarrier for Natural Drug Delivery through Blood-Brain Barrier. Adv. Pharmacol. Sci..

[280] Xie H., Li L., Sun Y., Wang Y., Gao S., Tian Y., Ma X., Guo C., Bo F., Zhang L. (2019). An Available Strategy for Nasal Brain Transport of Nanocomposite Based on PAMAM Dendrimers via In Situ Gel. Nanomaterials.

[281] Lee Y., Lee J., Kim M., Kim G.Y., Choi J.S., Lee M. (2021). Brain Gene Delivery Using Histidine and Arginine-Modified Dendrimers for Ischemic Stroke Therapy. J. Control. Release.

[282] Sharma A., Porterfield J.E., Smith E., Sharma R., Kannan S., Kannan R.M. (2018). Effect of Mannose Targeting of Hydroxyl PAMAM Dendrimers on Cellular and Organ Biodistribution in a Neonatal Brain Injury Model. J. Control. Release.

[283] Santos S.D., Xavier M., Leite D.M., Moreira D.A., Custódio B., Torrado M., Castro R., Leiro V., Rodrigues J., Tomás H. (2018). PAMAM Dendrimers: Blood-Brain Barrier Transport and Neuronal Uptake after Focal Brain Ischemia. J. Control. Release.

[284] Zhu Y., Liu C., Pang Z. (2019). Dendrimer-Based Drug Delivery Systems for Brain Targeting. Biomolecules.

[285] Moscariello P., Raabe M., Liu W., Bernhardt S., Qi H., Kaiser U., Wu Y., Weil T., Luhmann H.J., Hedrich J. (2019). Unraveling In Vivo Brain Transport of Protein-Coated Fluorescent Nanodiamonds. Small.

[286] Davoudi Z., Peroutka-Bigus N., Bellaire B., Jergens A., Wannemuehler M., Wang Q. (2021). Gut Organoid as a New Platform to Study Alginate and Chitosan Mediated PLGA Nanoparticles for Drug Delivery. Mar. Drugs.

[287] Zhi K., Raji B., Nookala A.R., Khan M.M., Nguyen X.H., Sakshi S., Pourmotabbed T., Yallapu M.M., Kochat H., Tadrous E. (2021). PLGA Nanoparticle-Based Formulations to Cross the Blood–Brain Barrier for Drug Delivery: From R&D to CGMP. Pharmaceutics.

[288] Li Y., Yin K., Diao Y., Fang M., Yang J., Zhang J., Cao H., Liu X., Jiang J. (2022). A Biopolymer-Gated Ionotronic Junctionless Oxide Transistor Array for Spatiotemporal Pain-Perception Emulation in Nociceptor Network. Nanoscale.

[289] Ghitman J., Biru E.I., Stan R., Iovu H. (2020). Review of Hybrid PLGA Nanoparticles: Future of Smart Drug Delivery and Theranostics Medicine. Mater. Des..

[290] Qu Z.S., Li L., Sun X.J., Zhao Y.W., Zhang J., Geng Z., Fu J.L., Ren Q.G. (2014). Glycogen Synthase Kinase-3 Regulates Production of Amyloid- β Peptides and Tau Phosphorylation in Diabetic Rat Brain. Sci. World J..

[291] Li C., Li W., Liu H., Zhang Y., Chen G., Li Z., Wang Q. (2020). An Activatable NIR-II Nanoprobe for In Vivo Early Real-Time Diagnosis of Traumatic Brain Injury. Angew. Chem. Int. Ed..

[292] Rabanel J.M., Piec P.A., Landri S., Patten S.A., Ramassamy C. (2020). Transport of PEGylated-PLA Nanoparticles across a Blood Brain Barrier Model, Entry into Neuronal Cells and in Vivo Brain Bioavailability. J. Control Release.

[293] Ma X., Aravind A., Pfister B.J., Chandra N., Haorah J. (2019). Animal Models of Traumatic Brain Injury and Assessment of Injury Severity. Mol. Neurobiol..

[294] Bouthour W., Mégevand P., Donoghue J., Lüscher C., Birbaumer N., Krack P. (2019). Biomarkers for Closed-Loop Deep Brain Stimulation in Parkinson Disease and Beyond. Nat. Rev. Neurol..

[295] Ledig C., Schuh A., Guerrero R., Heckemann R.A., Rueckert D. (2018). Structural Brain Imaging in Alzheimer’s Disease and Mild Cognitive Impairment: Biomarker Analysis and Shared Morphometry Database. Sci. Rep..

[296] Abdelmalik P.A., Draghic N., Ling G.S.F. (2019). Management of Moderate and Severe Traumatic Brain Injury. Transfusion.

[297] Shi K., Zhang J., Dong J.F., Shi F.D. (2019). Dissemination of Brain Inflammation in Traumatic Brain Injury. Cell. Mol. Immunol..

[298] Jarrahi A., Braun M., Ahluwalia M., Gupta R.v., Wilson M., Munie S., Ahluwalia P., Vender J.R., Vale F.L., Dhandapani K.M. (2020). Revisiting Traumatic Brain Injury: From Molecular Mechanisms to Therapeutic Interventions. Biomedicines.

[299] Yue J.K., Yuh E.L., Korley F.K., Winkler E.A., Sun X., Puffer R.C., Deng H., Choy W., Chandra A., Taylor S.R. (2019). Association between Plasma GFAP Concentrations and MRI Abnormalities in Patients with CT-Negative Traumatic Brain Injury in the TRACK-TBI Cohort: A Prospective Multicentre Study. Lancet Neurol..

[300] Maggio M.G., de Luca R., Molonia F., Porcari B., Destro M., Casella C., Salvati R., Bramanti P., Calabro R.S. (2019). Cognitive Rehabilitation in Patients with Traumatic Brain Injury: A Narrative Review on the Emerging Use of Virtual Reality. J. Clin. Neurosci..

[301] O’Brien W.T., Pham L., Symons G.F., Monif M., Shultz S.R., McDonald S.J. (2020). The NLRP3 Inflammasome in Traumatic Brain Injury: Potential as a Biomarker and Therapeutic Target. J. Neuroinflammation.

[302] Wang K.K., Yang Z., Zhu T., Shi Y., Rubenstein R., Tyndall J.A., Manley G.T. (2018). An Update on Diagnostic and Prognostic Biomarkers for Traumatic Brain Injury. Expert Rev. Mol. Deagn..

[303] Needham E.J., Helmy A., Zanier E.R., Jones J.L., Coles A.J., Menon D.K. (2019). The Immunological Response to Traumatic Brain Injury. J. Neuroimmunol..

[304] Gan Z.S., Stein S.C., Swanson R., Guan S., Garcia L., Mehta D., Smith D.H. (2019). Blood Biomarkers for Traumatic Brain Injury: A Quantitative Assessment of Diagnostic and Prognostic Accuracy. Front. Neurol..

[305] Ruozi B., Belletti D., Sharma H.S., Sharma A., Muresanu D.F., Mössler H., Forni F., Vandelli M.A., Tosi G. (2015). PLGA Nanoparticles Loaded Cerebrolysin: Studies on Their Preparation and Investigation of the Effect of Storage and Serum Stability with Reference to Traumatic Brain Injury. Mol. Neurobiol..

[306] Bailey Z.S., Nilson E., Bates J.A., Oyalowo A., Hockey K.S., Sajja V.S.S.S., Thorpe C., Rogers H., Dunn B., Frey A.S. (2020). Cerium Oxide Nanoparticles Improve Outcome after In Vitro and In Vivo Mild Traumatic Brain Injury. J. Neurotrauma.

[307] Pavlovic D., Pekic S., Stojanovic M., Popovic V. (2019). Traumatic Brain Injury: Neuropathological, Neurocognitive and Neurobehavioral Sequelae. Pituitary.

[308] Sharma M., Sharma R., Jain D.K., Saraf A. (2019). Enhancement of Oral Bioavailability of Poorly Water Soluble Carvedilol by Chitosan Nanoparticles: Optimization and Pharmacokinetic Study. Int. J. Biol. Macromol..

[309] Razzino C.A., Serafín V., Gamella M., Pedrero M., Montero-Calle A., Barderas R., Calero M., Lobo A.O., Yáñez-Sedeño P., Campuzano S. (2020). An Electrochemical Immunosensor Using Gold Nanoparticles-PAMAM-Nanostructured Screen-Printed Carbon Electrodes for Tau Protein Determination in Plasma and Brain Tissues from Alzheimer Patients. Biosens. Bioelectron..

[310] van Giau V., Bagyinszky E., Yang Y.S., Youn Y.C., An S.S.A., Kim S.Y. (2019). Genetic Analyses of Early-Onset Alzheimer’s Disease Using next Generation Sequencing. Sci. Rep..

[311] Wegmann S., Bennett R.E., Delorme L., Robbins A.B., Hu M., McKenzie D., Kirk M.J., Schiantarelli J., Tunio N., Amaral A.C. (2019). Experimental Evidence for the Age Dependence of Tau Protein Spread in the Brain. Sci. Adv..

[312] Ułamek-Kozioł M., Czuczwar S.J., Januszewski S., Pluta R. (2020). Proteomic and Genomic Changes in Tau Protein, Which Are Associated with Alzheimer’s Disease after Ischemia-Reperfusion Brain Injury. Int. J. Mol. Sci..

[313] Sundström A., Adolfsson A.N., Nordin M., Adolfsson R. (2020). Loneliness Increases the Risk of All-Cause Dementia and Alzheimer’s Disease. J. Gerontol. Ser. B.

[314] Monzio Compagnoni G., di Fonzo A., Corti S., Comi G.P., Bresolin N., Masliah E. (2020). The Role of Mitochondria in Neurodegenerative Diseases: The Lesson from Alzheimer’s Disease and Parkinson’s Disease. Mol. Neurobiol..

[315] Baik S.H., Kang S., Lee W., Choi H., Chung S., Kim J.I., Mook-Jung I. (2019). A Breakdown in Metabolic Reprogramming Causes Microglia Dysfunction in Alzheimer’s Disease. Cell Metab..

[316] Breijyeh Z., Karaman R., Muñoz-Torrero D., Dembinski R. (2020). Comprehensive Review on Alzheimer’s Disease: Causes and Treatment. Molecules.

[317] Uwishema O., Mahmoud A., Sun J., Correia I.F.S., Bejjani N., Alwan M., Nicholas A., Oluyemisi A., Dost B. (2022). Is Alzheimer’s Disease an Infectious Neurological Disease? A Review of the Literature. Brain Behav..

[318] Butterfield D.A., Halliwell B. (2019). Oxidative Stress, Dysfunctional Glucose Metabolism and Alzheimer Disease. Nat. Rev. Neurosci..

[319] Henstridge C.M., Hyman B.T., Spires-Jones T.L. (2019). Beyond the Neuron–Cellular Interactions Early in Alzheimer Disease Pathogenesis. Nat. Rev. Neurosci..

[320] dos Santos Tramontin N., da Silva S., Arruda R., Ugioni K.S., Canteiro P.B., de Bem Silveira G., Mendes C., Silveira P.C.L., Muller A.P. (2019). Gold Nanoparticles Treatment Reverses Brain Damage in Alzheimer’s Disease Model. Mol. Neurobiol..

[321] Sivanesan S., Rajeshkumar S. (2019). Gold Nanoparticles in Diagnosis and Treatment of Alzheimer’s Disease. Nanobiotechnology Neurodegener. Dis..

[322] Serafín V., Razzino C.A., Gamella M., Pedrero M., Povedano E., Montero-Calle A., Barderas R., Calero M., Lobo A.O., Yáñez-Sedeño P. (2020). Disposable Immunoplatforms for the Simultaneous Determination of Biomarkers for Neurodegenerative Disorders Using Poly(Amidoamine) Dendrimer/Gold Nanoparticle Nanocomposite. Anal. Bioanal. Chem..

[323] Monje M.H.G., Foffani G., Obeso J., Sánchez-Ferro A. (2019). New Sensor and Wearable Technologies to Aid in the Diagnosis and Treatment Monitoring of Parkinson’s Disease. Annu. Rev. Biomed. Eng..

[324] Byrom B., McCarthy M., Schueler P., Muehlhausen W. (2018). Brain Monitoring Devices in Neuroscience Clinical Research: The Potential of Remote Monitoring Using Sensors, Wearables, and Mobile Devices. Clin. Pharmacol. Ther..

[325] Kwon S., Kim H., Yeo W.H. (2021). Recent Advances in Wearable Sensors and Portable Electronics for Sleep Monitoring. iScience.

[326] Habets J.G.V., Heijmans M., Kuijf M.L., Janssen M.L.F., Temel Y., Kubben P.L. (2018). An Update on Adaptive Deep Brain Stimulation in Parkinson’s Disease. Mov. Disord..

[327] Romero L.E., Chatterjee P., Armentano R.L. (2016). An IoT Approach for Integration of Computational Intelligence and Wearable Sensors for Parkinson’s Disease Diagnosis and Monitoring. Health Technol..

[328] Yin R., Wang D., Zhao S., Lou Z., Shen G. (2021). Wearable Sensors-Enabled Human–Machine Interaction Systems: From Design to Application. Adv. Funct. Mater..

[329] Reddy V.S., Tian Y., Zhang C., Ye Z., Roy K., Chinnappan A., Ramakrishna S., Liu W., Ghosh R. (2021). A Review on Electrospun Nanofibers Based Advanced Applications: From Health Care to Energy Devices. Polymers.

[330] Reddy V.S., Agarwal B., Ye Z., Zhang C., Roy K., Chinnappan A., Narayan R.J., Ramakrishna S., Ghosh R. (2022). Recent Advancement in Biofluid-Based Glucose Sensors Using Invasive, Minimally Invasive, and Non-Invasive Technologies: A Review. Nanomaterials.

[331] Ghosh R., Pin K.Y., Reddy V.S., Jayathilaka W.A.D.M., Ji D., Serrano-García W., Bhargava S.K., Ramakrishna S., Chinnappan A. (2020). Micro/Nanofiber-Based Noninvasive Devices for Health Monitoring Diagnosis and Rehabilitation. Appl. Phys. Rev..

[332] Ashammakhi N., Hasan A., Kaarela O., Byambaa B., Sheikhi A., Gaharwar A.K., Khademhosseini A. (2019). Advancing Frontiers in Bone Bioprinting. Adv. Healthc. Mater..

[333] Ravanbakhsh H., Karamzadeh V., Bao G., Mongeau L., Juncker D., Zhang Y.S. (2021). Emerging Technologies in Multi-Material Bioprinting. Adv. Mater..

[334] Wang Y., Guo X., Li L.H., Zhang J., Li G.K., Zavabeti A., Li Y. (2022). Enhanced Piezoelectric Properties Enabled by Engineered Low-Dimensional Nanomaterials. ACS Appl. Nano. Mater..

[335] Lee E.K., Yoo H. (2021). Self-Powered Sensors: New Opportunities and Challenges from Two-Dimensional Nanomaterials. Molecules.

[336] Jayathilaka W.A.D.M., Qi K., Qin Y., Chinnappan A., Serrano-García W., Baskar C., Wang H., He J., Cui S., Thomas S.W. (2019). Significance of Nanomaterials in Wearables: A Review on Wearable Actuators and Sensors. Adv. Mater..

[337] Levato R., Visser J., Planell J.A., Engel E., Malda J., Mateos-Timoneda M.A. (2014). Biofabrication of Tissue Constructs by 3D Bioprinting of Cell-Laden Microcarriers. Biofabrication.

[338] Murphy S.v., Atala A. (2014). 3D Bioprinting of Tissues and Organs. Nat. Biotechnol..

[339] Dey M., Ozbolat I.T. (2020). 3D Bioprinting of Cells, Tissues and Organs. Sci. Rep..

[340] Decante G., Costa J.B., Silva-Correia J., Collins M.N., Reis R.L., Oliveira J.M. (2021). Engineering Bioinks for 3D Bioprinting. Biofabrication.

[341] Mandrycky C., Wang Z., Kim K., Kim D.H. (2016). 3D Bioprinting for Engineering Complex Tissues. Biotechnol. Adv..

[342] Zhu W., Qu X., Zhu J., Ma X., Patel S., Liu J., Wang P., Lai C.S.E., Gou M., Xu Y. (2017). Direct 3D Bioprinting of Prevascularized Tissue Constructs with Complex Microarchitecture. Biomaterials.

[343] Buchroithner B., Hartmann D., Mayr S., Oh Y.J., Sivun D., Karner A., Buchegger B., Griesser T., Hinterdorfer P., Klar T.A. (2020). 3D Multiphoton Lithography Using Biocompatible Polymers with Specific Mechanical Properties. Nanoscale Adv..

[344] Pardo A., Bakht S.M., Gomez-Florit M., Rial R., Monteiro R.F., Teixeira S.P.B., Taboada P., Reis R.L., Domingues R.M.A., Gomes M.E. (2022). Magnetically-Assisted 3D Bioprinting of Anisotropic Tissue-Mimetic Constructs. Adv Funct Mater.

[345] Mao S., Pang Y., Liu T., Shao Y., He J., Yang H., Mao Y., Sun W. (2020). Bioprinting of in Vitro Tumor Models for Personalized Cancer Treatment: A Review. Biofabrication.

[346] Marín-Morales J., Higuera-Trujillo J.L., Greco A., Guixeres J., Llinares C., Scilingo E.P., Alcañiz M., Valenza G. (2018). Affective Computing in Virtual Reality: Emotion Recognition from Brain and Heartbeat Dynamics Using Wearable Sensors. Sci. Rep..

[347] Hsieh F.Y., Hsu S.-h. (2016). 3D Bioprinting: A New Insight into the Therapeutic Strategy of Neural Tissue Regeneration. Organogenesis.

[348] Yoo J., Kim H.S., Hwang D.Y. (2013). Stem Cells as Promising Therapeutic Options for Neurological Disorders. J. Cell Biochem..

[349] Tasnim N., de la Vega L., Anil Kumar S., Abelseth L., Alonzo M., Amereh M., Joddar B., Willerth S.M. (2018). 3D Bioprinting Stem Cell Derived Tissues. Cell. Mol. Bioeng..

[350] Lee S.J., Esworthy T., Stake S., Miao S., Zuo Y.Y., Harris B.T., Zhang L.G. (2018). Advances in 3D Bioprinting for Neural Tissue Engineering. Adv. Biosyst..

[351] Alexander Heinrich M., Bansal R., Lammers T., Shrike Zhang Y., Michel Schiffelers R., Prakash J., Heinrich M.A., Bansal R., Prakash J., Lammers T. (2019). 3D-Bioprinted Mini-Brain: A Glioblastoma Model to Study Cellular Interactions and Therapeutics. Adv. Mater..

[352] Ngo M.T., Harley B.A.C. (2021). Progress in Mimicking Brain Microenvironments to Understand and Treat Neurological Disorders. APL Bioeng..

[353] Wang X., Ao Q., Tian X., Fan J., Tong H., Hou W., Bai S. (2017). Gelatin-Based Hydrogels for Organ 3D Bioprinting. Polymers.

[354] Shin J., Lee Y., Li Z., Hu J., Park S.S., Kim K. (2022). Optimized 3D Bioprinting Technology Based on Machine Learning: A Review of Recent Trends and Advances. Micromachines.

[355] Ngo M.T., Harley B.A.C. (2019). Perivascular Signals Alter Global Gene Expression Profile of Glioblastoma and Response to Temozolomide in a Gelatin Hydrogel. Biomaterials.

[356] Tirella A., Liberto T., Ahluwalia A. (2012). Riboflavin and Collagen: New Crosslinking Methods to Tailor the Stiffness of Hydrogels. Mater. Lett..

[357] Han J., Kim D.S., Jang H., Kim H.R., Kang H.W. (2019). Bioprinting of Three-Dimensional Dentin–Pulp Complex with Local Differentiation of Human Dental Pulp Stem Cells. J. Tissue Eng..

[358] Wang X., Li X., Dai X., Zhang X., Zhang J., Xu T., Lan Q. (2018). Coaxial Extrusion Bioprinted Shell-Core Hydrogel Microfibers Mimic Glioma Microenvironment and Enhance the Drug Resistance of Cancer Cells. Colloids Surf. B Biointerfaces.

[359] Cha J., Kim P. (2017). Biomimetic Strategies for the Glioblastoma Microenvironment. Front. Mater..

[360] Nishiguchi A., Zhang H., Schweizerhof S., Schulte M.F., Mourran A., Möller M. (2020). 4D Printing of a Light-Driven Soft Actuator with Programmed Printing Density. ACS Appl. Mater. Interfaces.

[361] Li Q., Lin H., Wang O., Qiu X., Kidambi S., Deleyrolle L.P., Reynolds B.A., Lei Y. (2016). Scalable Production of Glioblastoma Tumor-Initiating Cells in 3 Dimension Thermoreversible Hydrogels. Sci. Rep..

[362] Tang M., Rich J.N., Chen S. (2021). Biomaterials and 3D Bioprinting Strategies to Model Glioblastoma and the Blood–Brain Barrier. Adv. Mater..

[363] Raphael B., Khalil T., Workman V.L., Smith A., Brown C.P., Streuli C., Saiani A., Domingos M. (2017). 3D Cell Bioprinting of Self-Assembling Peptide-Based Hydrogels. Mater. Lett..

[364] Boonstra E., de Kleijn R., Colzato L.S., Alkemade A., Forstmann B.U., Nieuwenhuis S. (2015). Neurotransmitters as Food Supplements: The Effects of GABA on Brain and Behavior. Front. Psychol..

[365] Pham T.H., Gardier A.M. (2019). Fast-Acting Antidepressant Activity of Ketamine: Highlights on Brain Serotonin, Glutamate, and GABA Neurotransmission in Preclinical Studies. Pharmacol. Ther..

